# Genome-wide identification of *SERK* genes in apple and analyses of their role in stress responses and growth

**DOI:** 10.1186/s12864-018-5342-1

**Published:** 2018-12-27

**Authors:** Liwei Zheng, Juanjuan Ma, Jiangping Mao, Sheng Fan, Dong Zhang, Caiping Zhao, Na An, Mingyu Han

**Affiliations:** College of Horticulture, Northwest Agriculture & Forestry University, Yangling, 712100 Shaanxi China

**Keywords:** Apple, SERK, Gene expression, Stress response, Plant growth

## Abstract

**Background:**

Somatic embryogenesis receptor-like kinases (SERKs) are leucine-rich repeat receptor-like kinases associated with various signaling pathways. These kinases have a relationship with stress signals, and they are also believed to be important for regulating plant growth. However, information about this protein family in apple is limited.

**Results:**

Twelve apple *SERK* genes distributed across eight chromosomes were identified. These genes clustered into three distinct groups in a phylogenetic analysis. All of the encoded proteins contained typical SERK domains. The chromosomal locations, gene/protein structures, synteny, promoter sequences, protein–protein interactions, and physicochemical characteristics of *MdSERK* genes were analyzed. Bioinformatics analyses demonstrated that gene duplications have likely contributed to the expansion and evolution of *SERK* genes in the apple genome. Six homologs of *SERK* genes were identified between apple and *Arabidopsis*. Quantitative real-time PCR analyses revealed that the *MdSERK* genes showed different expression patterns in various tissues. Eight *MdSERK* genes were responsive to stress signals, such as methyl jasmonate, salicylic acid, abscisic acid, and salt (NaCl). The application of exogenous brassinosteroid and auxin increased the growth and endogenous hormone contents of *Malus hupehensis* seedlings. The expression levels of seven *MdSERK* genes were significantly upregulated by brassinosteroid and auxin. In addition, several *MdSERK* genes showed higher expression levels in standard trees of ‘Nagafu 2’ (CF)/CF than in dwarf trees of CF/‘Malling 9’ (M.9), and in CF than in the spur-type bud mutation “Yanfu 6” (YF).

**Conclusion:**

This study represents the first comprehensive investigation of the apple *SERK* gene family. These data indicate that apple *SERKs* may function in adaptation to adverse environmental conditions and may also play roles in controlling apple tree growth.

**Electronic supplementary material:**

The online version of this article (10.1186/s12864-018-5342-1) contains supplementary material, which is available to authorized users.

## Background

Many cell-surface receptors have been identified in plants in recent years. The somatic embryogenesis receptor-like kinases (SERKs) are examples of cell-surface receptors that participate in plant defense responses and growth. SERKs belong to the leucine-rich repeat II receptor-like kinase (LRRII-RLK) group, and have been highly conserved during evolution [1–4]. The first *SERK* gene was identified in *Daucus carota*, and was shown to play an important role in the formation from of embryos from single somatic cells [5]. *SERK* genes have recently been identified in *Arabidopsis thaliana*, tomato, rice and *Brassica rapa* [6–9].

The subcellular localization of SERK proteins may provide important clues about their function. As receptors for diverse signals (i.e., brassinosteroid (BR) signals, flagellin signals, male sporogenesis, and Mi-1-mediated resistance to potato aphids), SERK proteins mainly localize in the cell membrane [10]. They contain seven conserved domains: a signal peptide (SP) domain; a leu-zipper (ZIP) domain; five Leu-rich repeat (LRR) domains; a Ser-Pro-rich (SPP) domain; a transmembrane (TM) domain; and a cytoplasmic Ser/Thr kinase domain adjacent to the C-terminal domain (CTD) [11]. These conserved domains have important biological functions. In cells, protein transport is mediated by the SP domain [12]; the ZIP domain is essential for specific binding of SERK proteins [7, 12]; the LRR domain is essential for proteins to combine with plasma membranes [13]; the SPP domain is involved in the interaction between proteins and cell walls [7]; the TM domain separates the intracellular and extracellular regions of SERK proteins [14]; the cytoplasmic Ser/Thr kinase domain is related to the phosphorylation state of SERK proteins [13]; and the CTD controls the modification of mRNA precursors [15].

The plant *SERK* genes form small families. For example, there are five SERK proteins in *A. thaliana* and four close homologs in rice. However, they have various functions in processes ranging from embryo formation to fertility, defense responses against pathogens and fungi, abiotic stress resistance, and senescence [5, 7, 16, 17]. *AtSERK1* mediates ovule and embryo development and enhances embryogenic competence in culture [18]. *AtSERK2* functions as important control point for sporophytic development and affects male gametophyte production [19]. Some *SERK* genes, including *OsSERK1–2* and *AtSERK3–4,* take part in various stress and cell-death processes. *OsSERK1* can be activated by blast fungus, host cell death, defense signaling molecules, and other stress signals [6]. OsSERK2 positively regulates immunity in many signaling pathways [20]. *AtSERK3* is involved in the containment of dead cells after microbial infection [21], and *AtSERK4* functions redundantly with *AtSERK3* to regulate a BR-independent cell-death pathway [22].

*AtSERK3–4* and *AtSERK5-Ler* mediate BR signals, and are essential for normal plant growth and development. Brassinosteroid signaling shares close relationships with the signaling pathways of other hormones, including gibberellin (GA), auxin, cytokinin, and abscisic acid (ABA) [23–28]. Plant height and stem growth are mainly regulated by BR, and BR cannot perform this function without *AtSERK3–5* mediating the signal transduction pathway. In previous studies, overexpression of *AtSERK3/BAK1* or *AtSERK4/BKK1* caused stem elongation, while the *AtSERK3/BAK1* or *AtSERK4/BKK1* null mutation led to a semi-dwarf phenotype and reduced sensitivity to BR [29]; in *BR insensitive1–5* (*bri1–5*) and *serk3serk4* mutants, overexpressing *AtSERK5-Ler* with an intact RD motif restored the normal plant phenotype [28].

Since those studies, the important roles of *SERK* genes in stress responses and growth have been demonstrated in model plants. A few studies have focused on the roles of BR in apple. For instance, BR probably regulates apple seedling size and root formation [30]. Digital gene expression (DGE) analyses have shown that the dwarf phenotype of autotetraploid apple plants is related to the BR signaling pathway [31]; and BR was shown to stimulate the elongation of apple branches in vivo and to take part in mediating apple tree architecture [32, 33]. However, there is little information about *SERK* genes, which encode important BR signal receptors, in apple, and there have been no thorough and systematic studies on apple *SERK* genes.

Apple is an important perennial woody fruit crop in temperate regions. High stress resistance and vigorous vegetative growth of apple nursery trees are important attributes to apple producers and breeders. Abiotic stress can impact fruit yield and quality, and inappropriate vegetative growth suppresses early flowering and reduces yields in apple. Most widely grown apple cultivars are sensitive to adverse conditions and exhibit undesirable growth under such conditions, which is problematic for the apple industry. These two important problems are mediated by complex biological processes, including stress responses and hormone regulation. Therefore, the functional identification of apple *SERK* genes is very important.

To identify available information about the possible roles of *SERK* genes apple, we conducted a genome-wide search for apple *SERK* genes. The chromosomal location, gene/protein structure, evolutionary relationships, synteny, promoter sequences, protein–protein interactions, physicochemical characteristics, and expression profiles of these genes were also analyzed. We determined the expression patterns of *MdSERK* genes in response to stress signals, BR and auxin treatments, and in different graft combinations and apple varieties. The results of this study not only provide insights into *SERKs* but also form the basis for further studies on their potential functions in apple.

## Results

### Genome-wide identification, distribution, and multiple sequence alignment of apple *SERK* genes

**Fig. 1:**
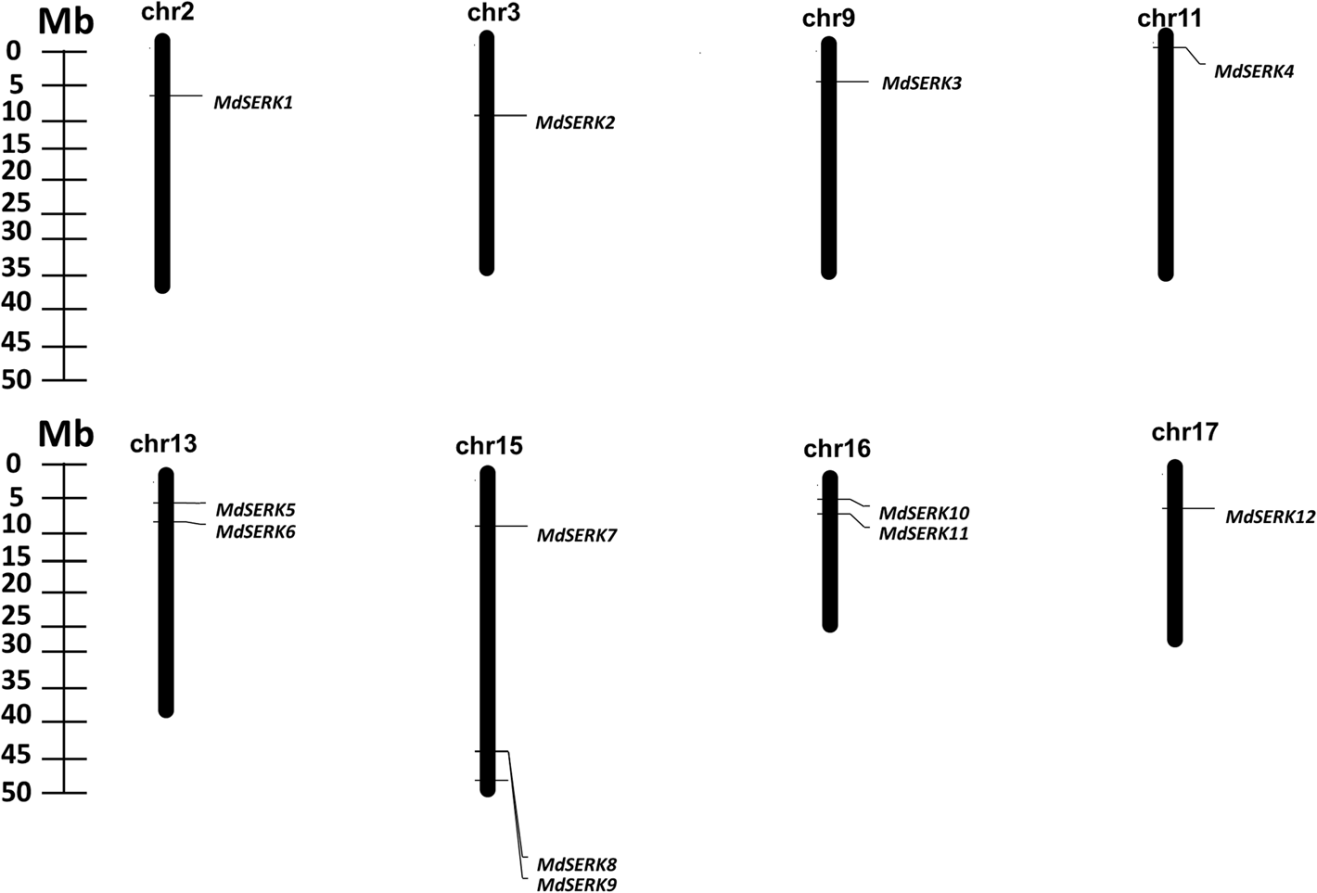
Chromosomal mapping of apple *SERK* gene family. Scale represents megabases (Mb). Chromosome numbers are indicated above each bar

In total, 12 candidate apple *SERK* genes were identified. According to their chromosomal locations, they were named *MdSERK1* to *MdSERK12.* Their deduced polypeptides ranged in length from 554 (*MdSERK4*) to 1274 (*MdSERK11*) amino acids (aa), and their predicted molecular weights ranged from 61.48 (*MdSERK4*) to 143.65 (*MdSERK11*) kDa (Table 1). The NCBI apple expressed sequence tag (EST) database was screened to evaluate the accuracy of the genomic predictions. All of the putative genes matched to at least one EST (Additional file 1: Table S2). Their protein sequences are shown in Additional file 1: Table S3. Protein sequences were analyzed with ExPASy to predict protein characteristics. Most of the MdSERK proteins were found to be stable with a low instability index (< 40), except for MdSERK1, MdSERK4, and MdSERK5. All MdSERK proteins were hydrophilic according to their grand average of hydropathicity (GRAVY) values. The predominant aa residues were Leu, Ser, and Gly. We also detected Ala and Val in the MdSERK proteins. The aliphatic index values ranged from 81.61 (MdSERK6) to 96.90 (MdSERK9). The predicted isoelectric point (pI) ranged from 5.45 (*MdSERK1*) to 8.69 (*MdSERK11*). Seven of the 12 MdSERK proteins comprised acidic amino acids and the others comprised basic amino acids. All MdSERK proteins were predicted to be localized in the cell membrane (Additional file 1: Table S4). The 12 genes were distributed across eight of the 17 *Malus* × *domestica* chromosomes (Fig. 1). The number of *MdSERK* genes ranged from one to three genes per chromosome. One *MdSERK* gene was detected on chromosomes 2, 3, 9, 11, and 17, and two *MdSERK* genes were detected on chromosomes 13 and 16. Chromosome 15 contained three *MdSERK* genes, the maximum number among all chromosomes (Fig. 1).

**Table 1:** Apple *SERK* genes identified in this study

Gene name	Gene locus	Chromosome no.	Start	End	Length (aa)	MW (kDa)
*MdSERK1*	MDP0000291093	chr2	7,625,329	7,632,528	627	69.00
*MdSERK2*	MDP0000935390	chr3	11,115,292	11,118,847	638	69.72
*MdSERK3*	MDP0000031416	Chr9	4,860,518	4,863,568	623	68.22
*MdSERK4*	MDP0000432466	chr11	741,533	748,228	554	61.48
*MdSERK5*	MDP0000211724	chr13	3,346,698	3,349,775	586	65.02
*MdSERK6*	MDP0000252094	chr13	6,351,809	6,360,764	1254	140.26
*MdSERK7*	MDP0000131814	chr15	7,182,082	7,186,127	590	66.51
*MdSERK8*	MDP0000287771	chr15	42,916,747	42,924,541	947	105.32
*MdSERK9*	MDP0000309283	chr15	42,918,529	42,925,637	774	85.79
*MdSERK10*	MDP0000887896	chr16	2,216,029	2,219,238	625	68.95
*MdSERK11*	MDP0000196862	chr16	4,655,977	4,664,288	1274	143.65
*MdSERK12*	MDP0000202785	chr17	5,408,191	5,411,316	585	64.33

^a^Gene locus corresponds to annotated ID from apple (*Malus* × *domestica*) genome data

**Fig. 2:**
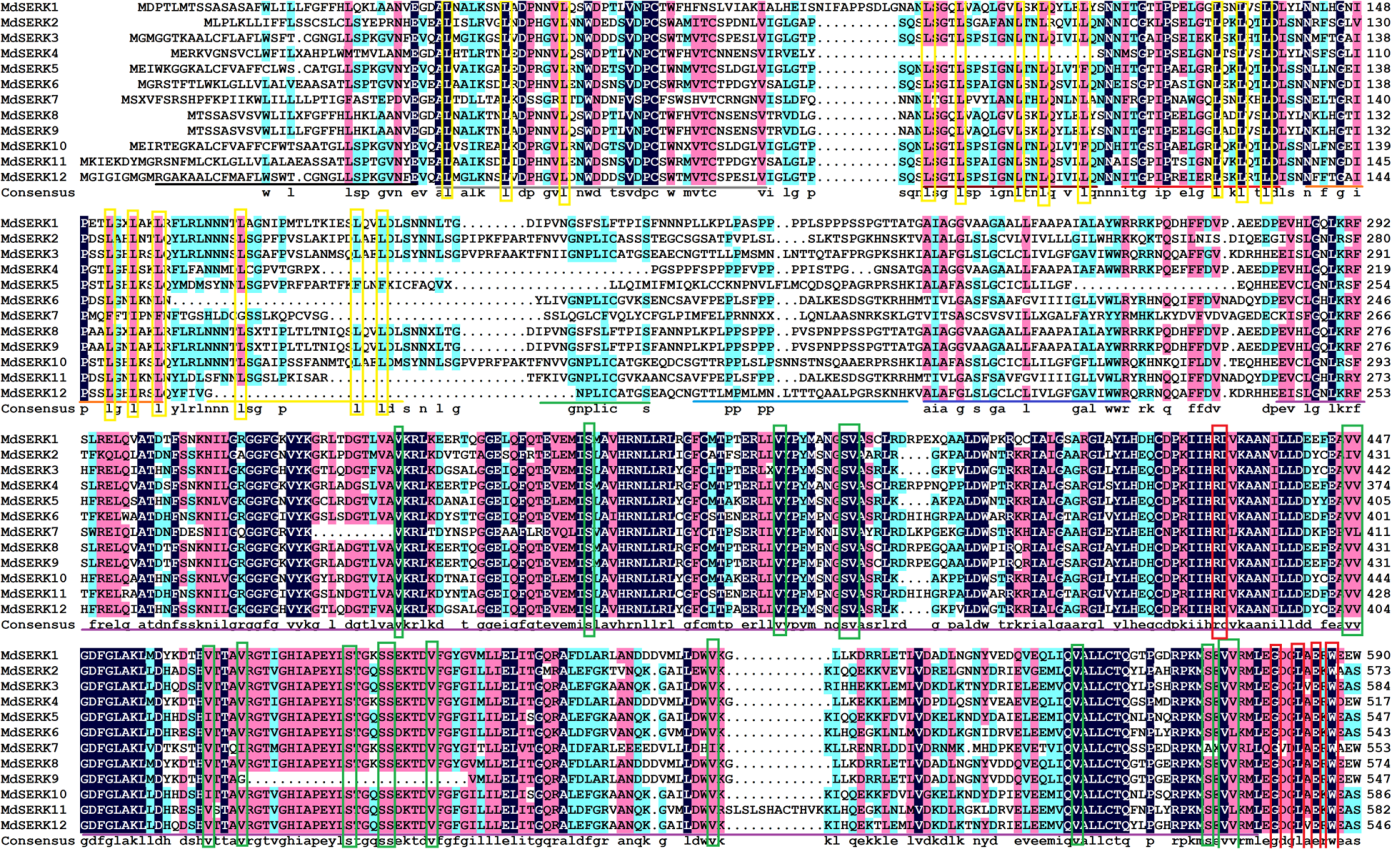
Alignment of multiple apple SERK sequences. Conserved sequence characteristics of MdSERKs are indicated with colored underlines (black, signal peptide; gray, leucine zipper; deep red, red, orange, yellow, and green, leucine-rich repeat 1–5, respectively; dark green, serine-proline-rich domain; indigo, transmembrane domain; purple, kinase domain; brown, C-terminal domain). Conserved amino acid residues and RD motif are indicated with colored rectangles

Details of domain structures were obtained from multiple sequence alignments (Fig. 2). The *MdSERK* genes encoded proteins with the expected SERK domains, including (from the N to C termini) the SP domain, the ZIP domain, five LRR domains, the SPP domain, the TM domain, the cytoplasmic Ser/Thr kinase domain, and the CTD (Fig. 2). In the encoded SERKs, the N-terminal SP aa residues were variable except for Leu, Asp, and Glu residues. With only minor sequence variations, the Leu residues of the Leu zipper (highlighted with a yellow box) were essentially conserved. The Leu residues of three of the five Leu-rich repeats (all except for LRR4 and LRR5) were also conserved (highlighted with a yellow box). The SPP and TM domains were not highly conserved. In contrast, the Ser/Thr kinase domain was essentially conserved at Ser and Val (green box) and it also shared a conserved RD motif (red box). The C-terminal domain comprised about 20 aa and had conserved Gly, Leu, Glu, and Trp residues (red box) (Fig. 2). Ten consensus motifs were identified using the MEME motif search tool (Additional file 2: Figure S1; Additional file 1: Table S5). Based on location information, the kinase domain consisted of motifs 1–5 and 8–10; the Leu zipper contained motifs 6 and 7; and the LRR domain included motif 6. The predicted protein structures analysis showed that all MdSERK proteins consisted of α helices, β turns, extended strands, and random coils (Additional file 3: Figure S2, Additional file 1: Table S6). Among these structures, α helices were the most abundant and largest, while β turns were the least abundant and smallest. Random coils were larger than extended strands both in terms of amino acid length and proportion. The transmembrane helices of all MdSERK proteins were also analyzed. At least one transmembrane segment was detected in all MdSERK proteins, except for MdSERK5 (Additional file 4: Figure S3). There was one transmembrane segment in MdSERK2–3, MdSERK6–8, and MdSERK10–12. There were two transmembrane segments in MdSERK1 and MdSERK9, and three in MdSERK4.

### Phylogenetic and gene structure analysis of apple *SERK* gene family

**Fig. 3:**
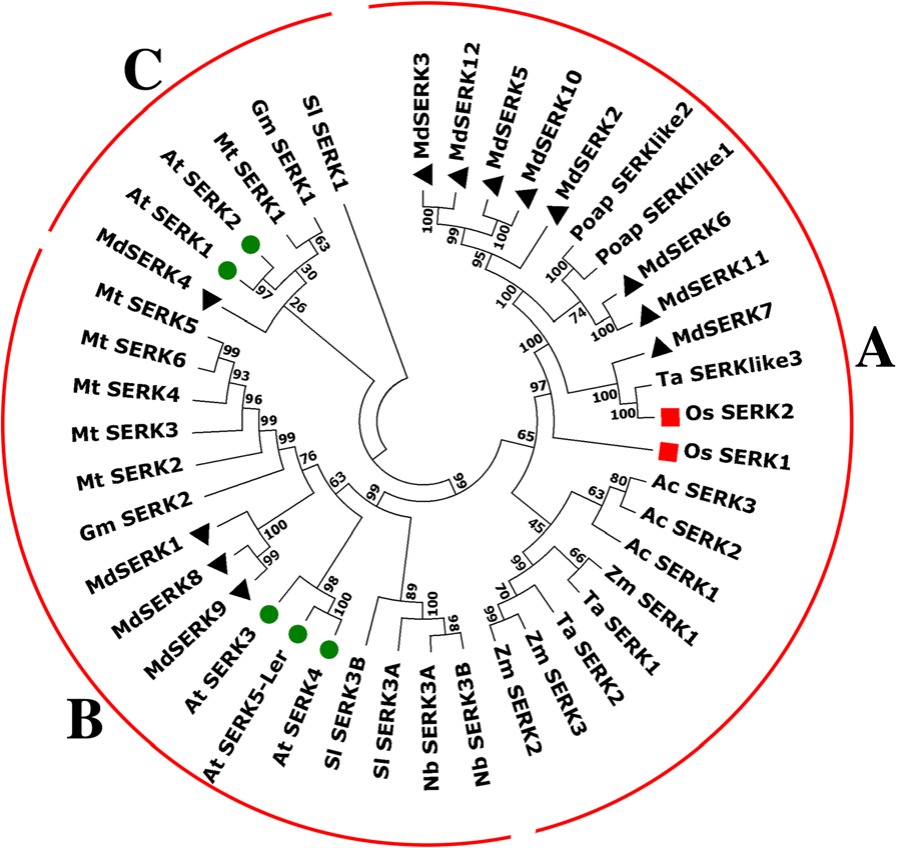
Phylogenetic analysis of SERKs from *Malus* × *domestica* and other plant species. Unrooted phylogenetic tree was constructed using the neighbor-joining method with MEGA 7. Apple, *Arabidopsis* and rice SERK proteins are indicated by black triangle, green circle, and red square, respectively

The amino acid sequences of SERKs from various plant species were compared to evaluate the evolutionary relationships among these kinases. Then, an unrooted phylogenetic tree was constructed to identify putative orthologs. In the tree, the tested species clustered into three distinct groups (i.e., A, B, and C) (Fig. 3).

As shown in Fig. 3, in Group A, *MdSERK1,* and *MdSERK8–9* clustered with *SISERK3A/B*, *NbSERK3A/B*, *MtSERK2–6, AtSERK3–4, AtSERK5-Ler,* and others. In Group B, *MdSERK4* was closely related to *AtSERK1–2, GmSERK1*, *MtSERK1*, and *SISERK1*, all of which were from different plant species. The remaining *MdSERK* genes clustered into Group C, which contained genes encoding OsSERK1–2, TaSERKlike3, and PoapSERK2 proteins. There were four sister pairs in apple: *MdSERK8/ MdSERK9, MdSERK6/MdSERK11, MdSERK5/MdSERK10,* and *MdSERK3/MdSERK12.*

An unrooted phylogenetic tree was constructed using the protein sequences encoded by the apple *SERK* genes (Additional file 5: Figure S4). Three gene categories were identified, similar to the phylogenetic groups discussed above. As expected, most *SERK* genes within the same group had very similar intron/exon distribution patterns in terms of exon length and intron number. Group A had seven members; *MdSERK2–3*, *MdSERK*5, *MdSERK10* and *MdSERK11.* These genes were similar in exon length and intron length, and *MdSERK6* shared similar intron phases with *MdSERK11*. Among Group B genes, *MdSERK8* and *MdSERK9* shared identical intron phases and exon numbers. Group C consisted of only one *MdSERK* gene with nine exons and eight introns.

### Synteny analysis, promoter analysis, and interaction network of apple *SERK* genes

**Fig. 4:**
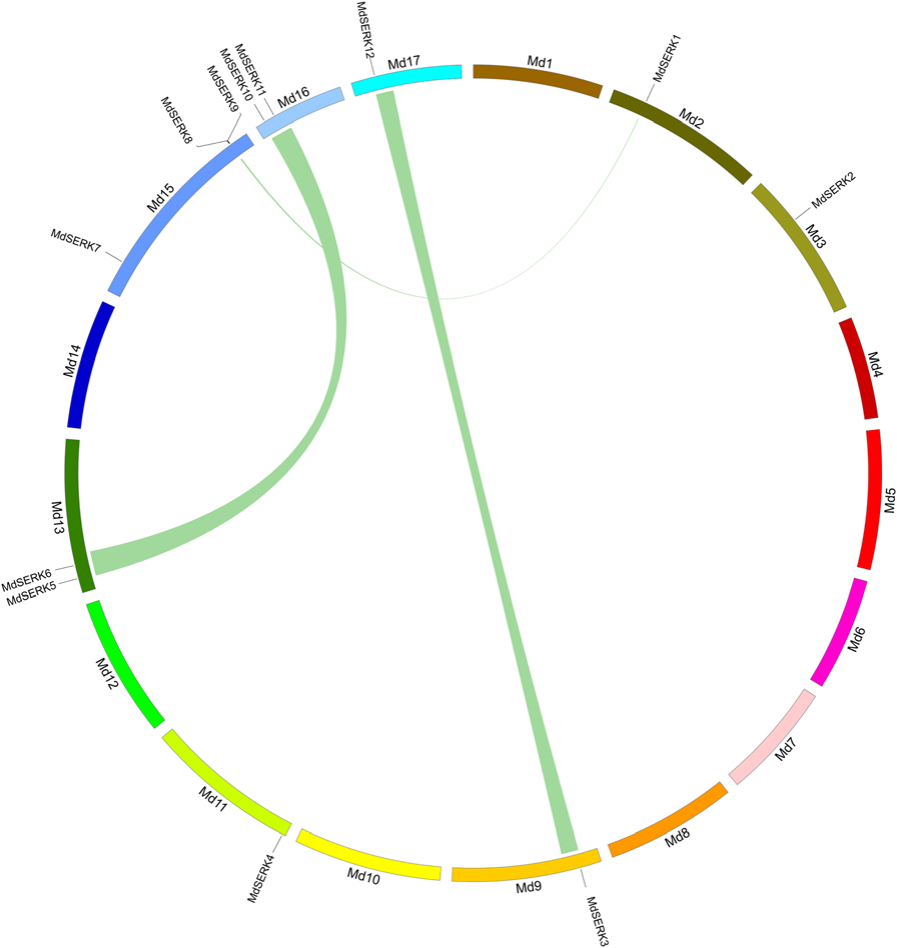
Synteny of *MdSERK* genes. Location of *MdSERK* genes on apple chromosomes (Md1–17). Syntenic regions in apple genome are connected by green lines

**Fig. 5:**
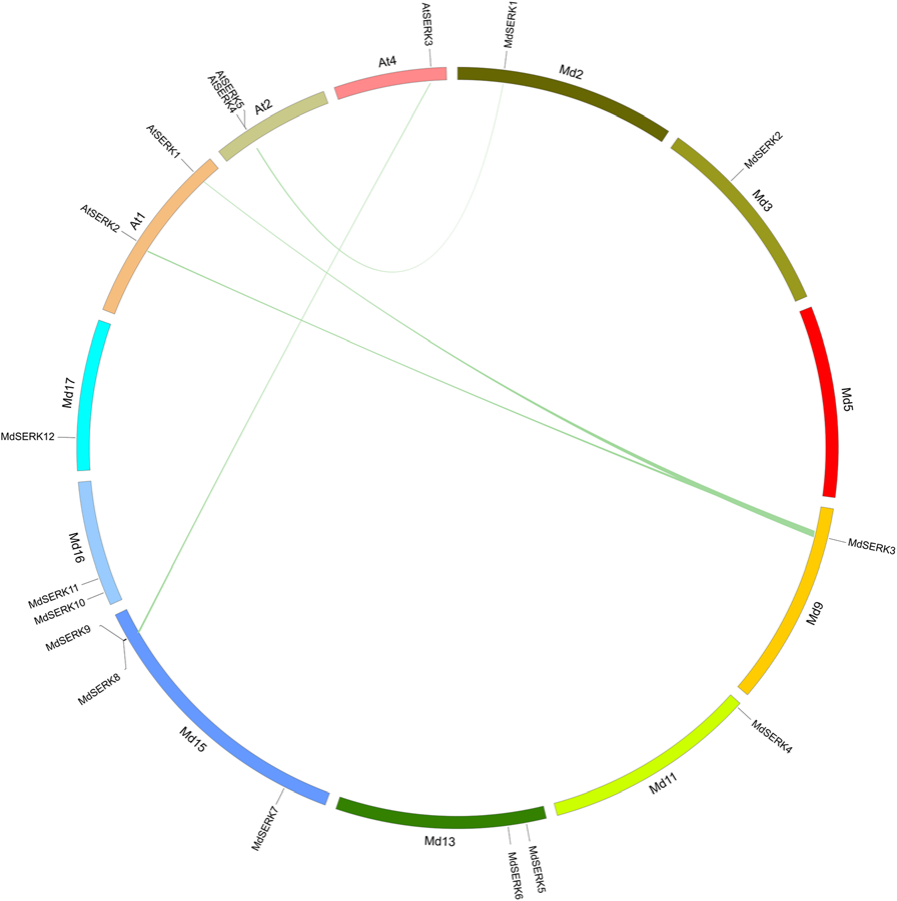
Synteny analysis of *SERK* genes between apple and *Arabidopsis*. All *MdSERK* and *AtSERK* genes in apple chromosomes and *Arabidopsis* chromosomes, respectively. Syntenic regions between apple and *Arabidopsis* chromosomes are connected by green lines

Because gene duplication events have occurred during the evolution of plant genomes, we searched for *MdSERK* duplicates. Segmental and tandem duplications were defined based on published criteria [34, 35]. One tandem duplication was identified: *MdSERK8* and *MdSERK9* on chromosome 15 (Fig. 4). The following five segmental duplications were also detected: *MdSERK1* (chromosome 2) and *MdSERK8/9* (chromosome 15); *MdSERK3* (chromosome 9) and *MdSERK12* (chromosome 17); *MdSERK5* (chromosome 13) and *MdSERK10* (chromosome 16); and *MdSERK6* (chromosome 13) and *MdSERK11* (chromosome 16) (Fig. 4; Additional file 1: Table S7). A syntenic map between *AtSERK* and *MdSERK* was generated (Fig. 5). In total, six *SERK* orthologs in *Arabidopsis* and apple were identified. *MdSERK1–AtSERK4/5, MdSERK3–AtSERK1/2,* and *MdSERK8/9–AtSERK3* were located in duplicated genomic regions between the apple and *Arabidopsis* genomes (Additional file 1: Table S8).

The promoter regions (about 1.5 kb) of *MdSERK* genes (Additional file 6: Figure S5) were found to contain important *cis*-acting elements associated with stress and hormone-related responses. At least one stress-specific activation element was present in the promoter of all *MdSERK* genes. There were one to three *cis*-acting salicylic acid (SA)-responsive (TCA) elements in all *MdSERK* genes, except for *MdSERK1, MdSERK3–4* and *MdSERK7;* one to two CGTCA motifs in all *MdSERK* genes; and one to three ABA-responsive elements (ABREs) in *MdSERK1–10*. These included the BR-response element G-box or non-E-box in all studied genes except for *MdSERK2* and *MdSERK8* [36]. There was more than one auxin-responsive element (TGA) in all *MdSERK* genes except for *MdSERK4, MdSERK7,* and *MdSERK8.* All genes contained more than one GA-response element (GARE) motif (i.e., one GARE-motif and a P-box in *MdSERK2,* two P-boxes in *MdSERK3,* and two GARE-motifs in *MdSERK5*) [37] in all other *MdSERK* genes).

An interaction network for the MdSERK proteins was constructed using *A. thaliana* orthologs (Additional file 7: Figure S6). Five of the 10 *MdSERK* genes were orthologs of two out of five *AtSERK* genes. The MdSERK partners mainly included BAK1/SERK3 and SERK4, which are involved in BR and flagellin signal transduction and in the regulation of stem elongation, leaf development, xylem differentiation, and cell-death responses. MdSERK1, MdSERK4, and MdSERK8–9, which were found to be highly homologous to AtSERK3/AtBAK1, formed relatively strong interactions with BRI1, BKI1, BSK1, BES1, and SERK4. MdSERK2–3 and MdSERK12, MdSERK5 and MdSERK10, and MdSERK6 and MdSERK11 were homologous to *A. thaliana* NSP-interacting kinases 1–3 (NIK1–3) which are involved in the defense response to geminivirus infection, and exhibited strong interactions with SUPPRESSOR OF ACAULIS 52 (SAC52), which is involved in translational regulation and plant height control.

### Apple *SERK* expression profiles in different tissues

**Fig. 6:**
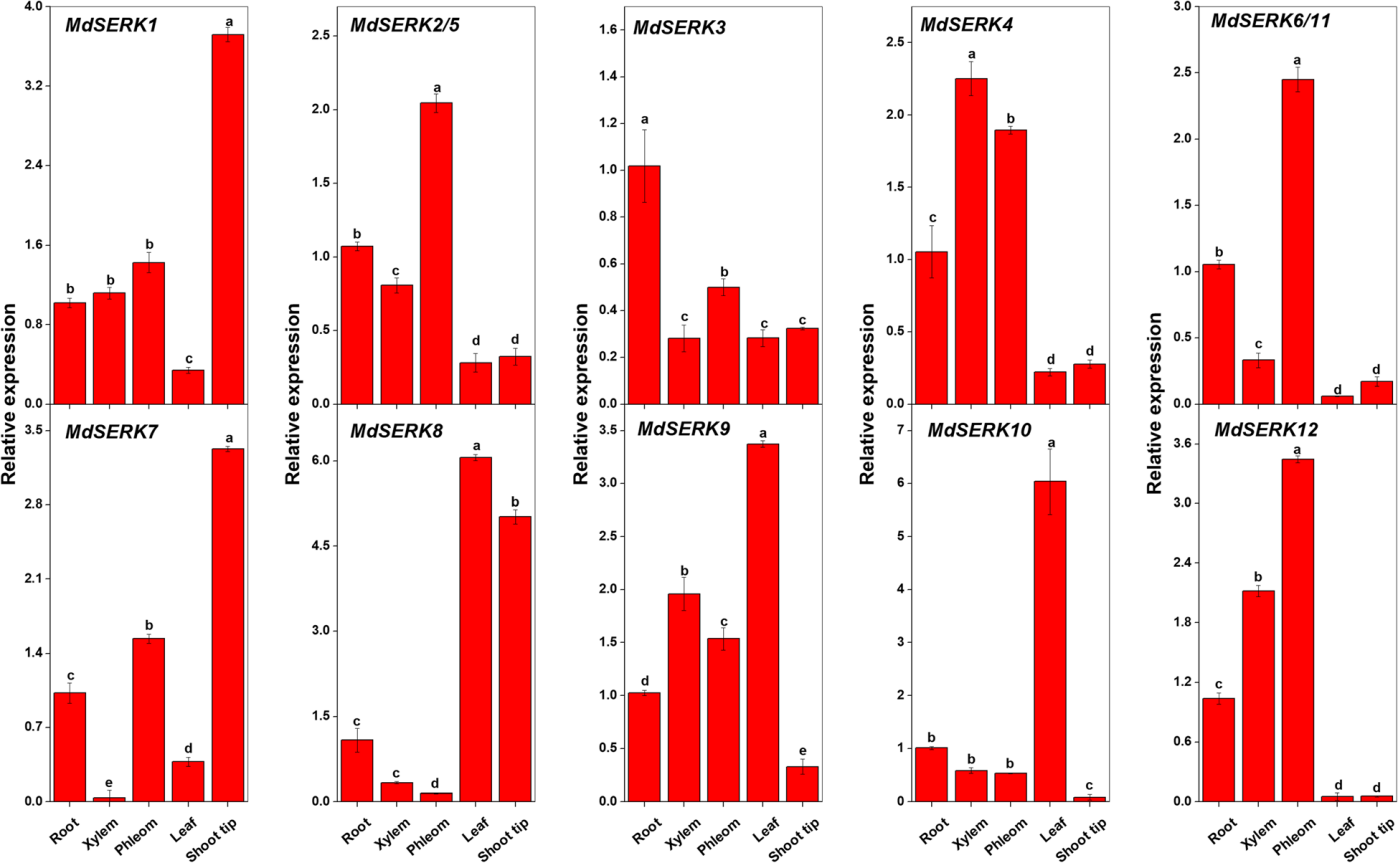
Expression profiles of apple *SERK* genes in different tissues. Quantitative reverse transcription polymerase chain reaction analysis of *SERK* gene expression levels in roots, xylem and phloem of stems, leaves, and shoot tips. Bars show mean ± standard error (*n* = 3). Overall least significant difference (*p* < 0.05) was calculated and used to separate means

The tissue-specific expression patterns of *MdSERK* genes were analyzed in various tissues of *M. hupehensis* seedlings by quantitative real-time polymerase chain reaction (qRT-PCR) (Fig. 6). *MdSERK1* and *MdSERK7* were highly expressed in shoot tips. *MdSERK2/5* and *MdSERK6/11* showed the highest expression levels in the phloem. The expression levels of *MdSERK4* and *MdSERK12* were higher in the xylem and phloem of the stem than in other tissues. *MdSERK3* was strongly expressed in the root, and the highest expression levels of *MdSERK8–10* were in the leaf.

To elucidate the potential roles and functions of the *MdSERK* genes in apple, expression profile data for different tissues (seedlings, roots, stems, flowers, fruit, and leaves) with two biological replicates were downloaded from the ArrayExpress database (E-GEOD-42873) (Additional file 8: Figure S7). Almost all *MdSERK* genes (except for *MdSERK8–9* and *MdSERK11*) were expressed at higher levels in the flower of M74, fruit of M20_100 daf (100 days after flowering) and M.20_harvest, leaf of M49, and fruit of M74_harvest than in other tissues. *MdSERK8–9* and *MdSERK11* showed low expression levels in almost all tissues, especially in seedlings of Golden delicious (GD), compared with the other *MdSERK* genes.

### Effects of exogenous hormone and salt treatments on gene expression profiles

**Fig. 7:**
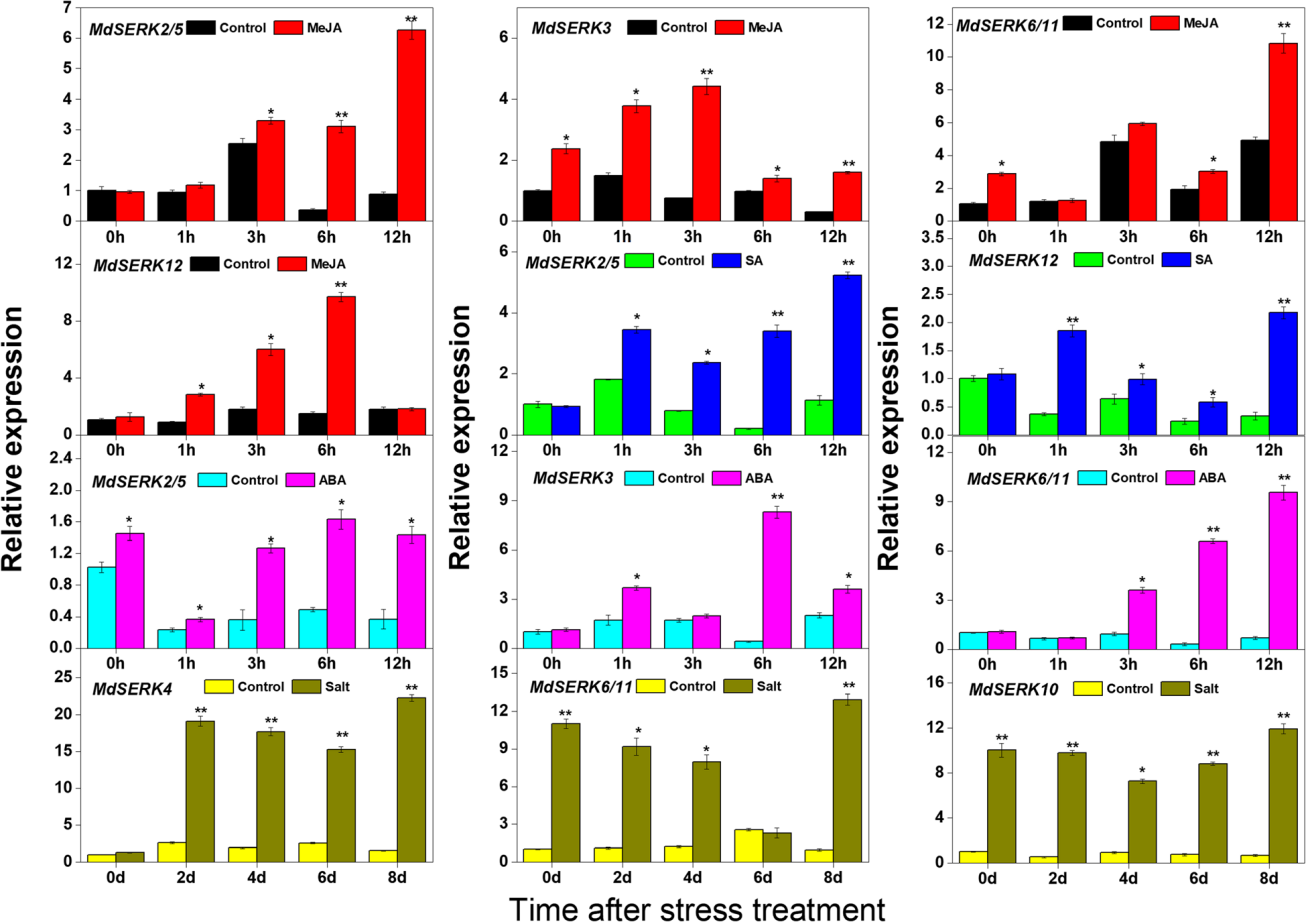
Transcript levels of *MdSERKs* after hormone and salt treatments. Quantitative reverse transcription polymerase chain reaction analysis of selected apple *SERK* genes in response to methyl jasmonate (MeJA), salicylic acid (SA), abscisic acid (ABA), and NaCl treatments at different time points (0, 1, 3, 6, and 12 h). *EF1-α* was used as an internal control. qRT-PCR data are shown relative to 0 h. Bars show mean ± standard error (*n* = 3). *Significant difference at 0.05 level, **significant difference at 0.01 level

To investigate the roles of *MdSERK* genes in stress responses, *MdSERK* gene expression patterns in young leaves were separately analyzed after methyl jasmonate (MeJA), SA, ABA, and salt treatments (Fig. 7). *MdSERK2/5, MdSERK3, MdSERK6/11,* and *MdSERK12* were induced by MeJA. The expression level of *MdSERK2/5* increased by 0.2- to 5-fold from 3 to 12 h in the MeJA-treated plants. *MdSERK3* expression increased by 1.3-, 1.5-, 4-, 0.3-, and 5-fold at 0, 1, 3, 6, and 12 h, respectively, after MeJA treatment. *MdSERK6/11* expression increased by about 2-, 0.5-, and 2.5-fold at 0, 6, and 12 h after MeJA treatment. The expression level of *MdSERK12* was increased by about 3-, 3.2-, and 6-fold at 1, 3, and 6 h after MeJA treatment. *MdSERK2/5* and *MdSERK12* expression levels were also increased by about 0.5- to 15-fold at most time points after SA treatment. *MdSERK2/5* and *MdSERK12* showed higher expression levels in all treatment groups than in control groups from 1 to 12 h. The transcript levels of *MdSERK2/5* were higher in the ABA treatment group than in the control group throughout the whole experiment. The relative expression level of *MdSERK3* was significantly increased at 1, 6, and 12 h after ABA treatment, and those of *MdSERK6/11* were upregulated by about 2-, 13-, and 9-fold at 3, 6, and 12 h after ABA treatment. The NaCl treatment increased the transcript levels of *MdSERK4, MdSERK6/11,* and *MdSERK10* in leaves. *MdSERK4* was induced by NaCl at all time points, except for 0 d. The transcript levels of *MdSERK6/11* in leaves were about 10-, 8-, 6.5-, and 11.5-fold higher in the NaCl-treated group than in the control group. After the NaCl treatment, *MdSERK10* was upregulated by about 7 to 12 times. The transcript levels of the other *MdSERK* genes were neither increased nor decreased in response to stress treatments (data not shown).

### Effects of exogenous BR and auxin treatments on apple growth, endogenous hormone contents, and gene expression profiles in *M. hupehensis* seedlings

**Fig. 8:**
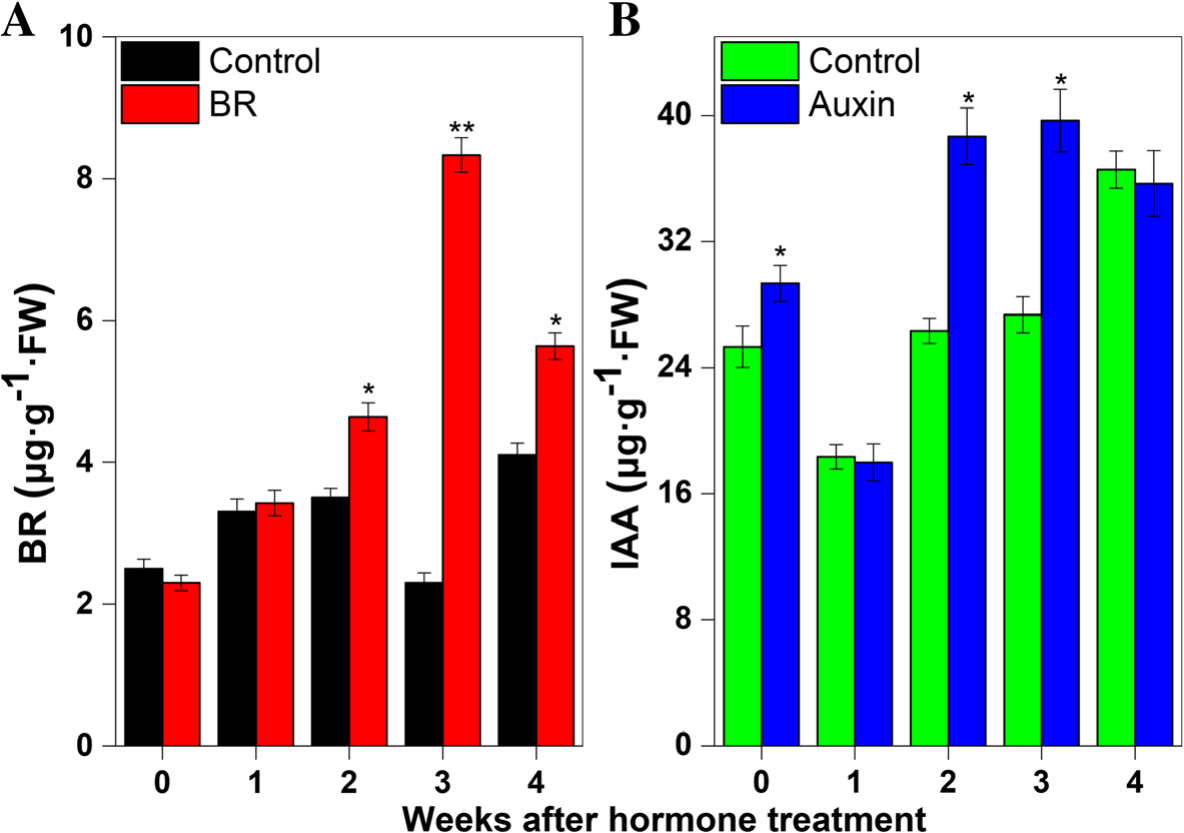
Endogenous hormone contents in shoot tips of control, brassinosteroid (BR)-treated, and auxin-treated plants. **a** Contents of BR in control and BR-treated groups. **b** Content of auxin in control and auxin-treated groups. Bars show mean ± standard error (*n* = 3). *Significant difference at 0.05 level, **significant difference at 0.01 level

**Fig. 9:**
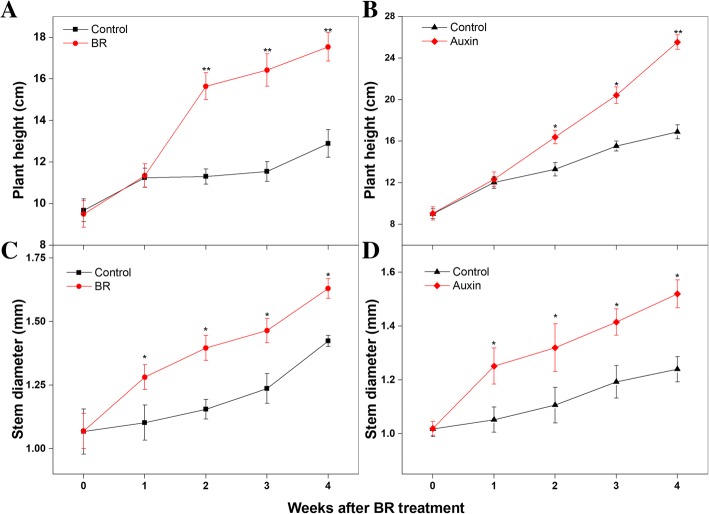
Height of *Malus hupehensis* seedlings after brassinosteroid (BR) and auxin treatments. Plant height was measured at 0, 1, 2, 3 and 4 weeks after BR (**a**) and auxin (**b**) treatments. Bars show mean ± standard error (*n* = 3). Stem diameter was measured at 0, 1, 2, 3 and 4 weeks after BR (**c**) and auxin (**d**) treatments. *Significant difference at 0.05 level, **Significant difference at 0.01 level

Endogenous BR and auxin were simultaneously analyzed after exogenous hormone applications (Fig. 8). The BR content in shoot tips was high from 2 to 4 weeks after BR treatment (Fig. 8a), and the level of auxin (indole-3-acetic acid, IAA) in the auxin-treated group increased to about 4, 12.3, and 12.4 μg·g^− 1^ FW at 0, 2, and 3 weeks, respectively, after the auxin treatment (Fig. 8b). The plant height and stem diameter of *M. hupehensis* seedlings were measured after treatments with BR and auxin. The plant height had increased significantly by 2, 3, and 4 weeks after hormone application (Fig. 9a and Fig. 9b). The plants in the BR treatment groups were about 4 cm taller than those in the control group at 4 weeks after treatment (Fig. 9a). The height increased by about 9 cm at 4 weeks after the auxin treatment (Fig. 9b). Compared with control plants, those treated with BR had a significantly greater stem diameter from 1 to 4 weeks after the treatment (Fig. 9c). Like the BR treatment, the auxin treatment increased the lateral growth of stems. Compared with control plants, the auxin-treated plants showed 0.2-, 0.21-, 0.22-, and 0.28-mm thicker stems at 1, 2, 3, and 4 weeks, respectively, after the auxin treatment (Fig. 9d).

**Fig. 10:**
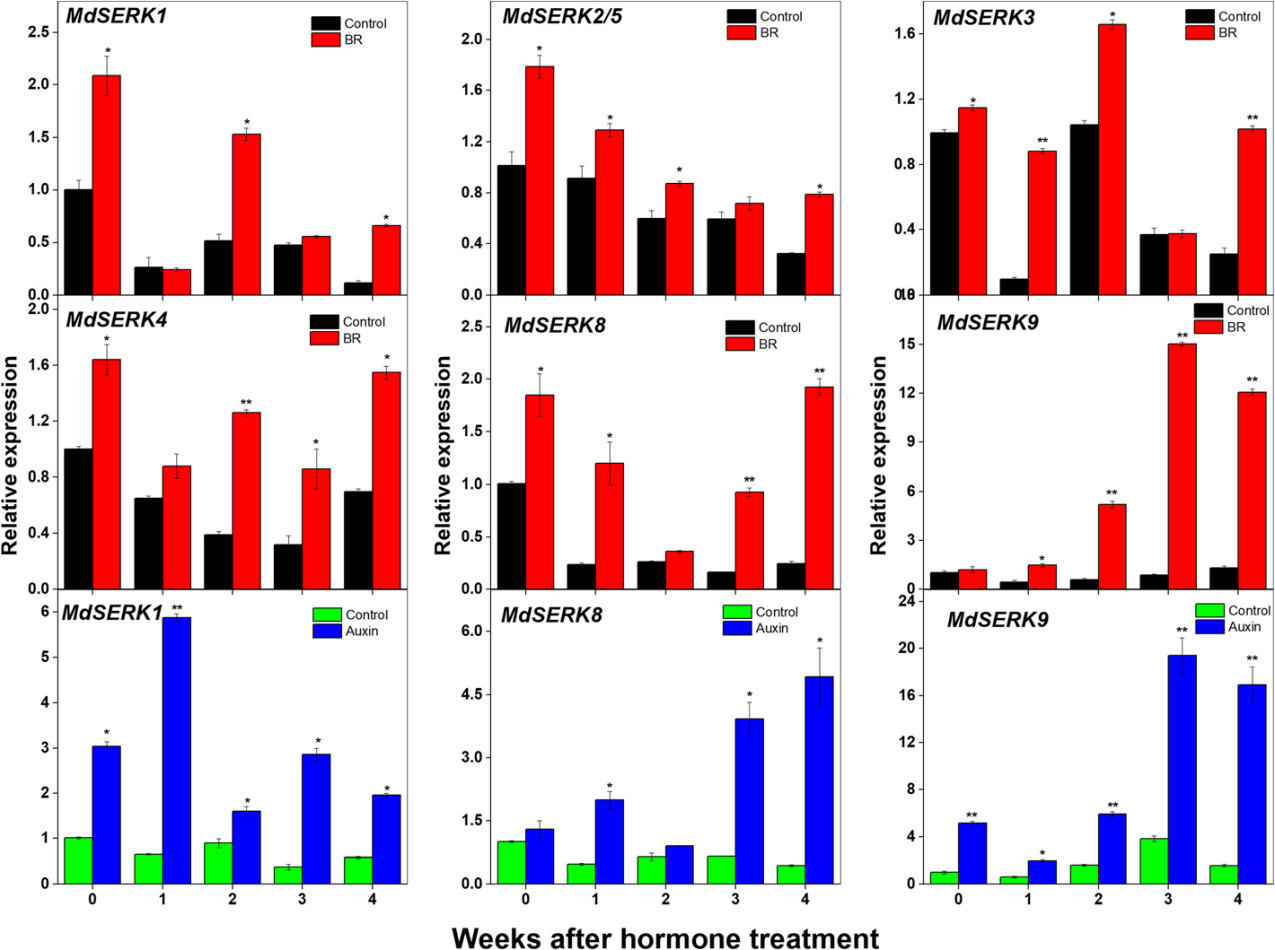
Expression of *SERK* genes in response to brassinosteroid (BR) and auxin treatments. Quantitative reverse transcription polymerase chain reaction analysis of apple *SERK* genes in response to BR and auxin treatments at different time points (0, 1, 2, 3, and 4 weeks). *EF1-α* was used as an internal control. qRT-PCR data are shown relative to 0 weeks. Bars show mean ± standard error (*n* = 3). *Significant difference at 0.05 level, **significant difference at 0.01 level

The *MdSERK* expression levels were evaluated in the shoot tips of *M. hupehensis* after the hormone treatments (Fig. 10). The transcript levels of several *MdSERK* genes were significantly upregulated at most time points after the BR treatment. For instance, the transcript level of *MdSERK1* was increased by about 1.2-, 2.1-, and 3-fold at 0, 2, and 4 weeks after the BR treatment. The transcript levels of *MdSERK2/5* and *MdSERK3* were increased by about 0.3- to 3-fold at 0, 1, 2, and 4 weeks after the BR treatment. The transcript level of *MdSERK4* was increased by 0.7- to 3-fold at 0, 2, 3, and 4 weeks after the BR treatment. *MdSERK8* was upregulated in BR-treated shoot tips compared with the control at all time points, except at 2 weeks. The *MdSERK9* expression levels were increased by approximately 2- to 15-fold, compared with that in the control, at most time points after the exogenous BR treatment.

*MdSERK1, MdSERK8,* and *MdSERK9* were significantly upregulated after the auxin treatment (Fig. 10). *MdSERK1* and *MdSERK9* were induced by approximately 0.6- to 15-fold after auxin treatment*,* and the transcript level of *MdSERK8* was increased by about 3- to 8-fold at 1, 3, and 4 weeks after the auxin treatment. The transcript levels of other *MdSERK* genes either increased or decreased at different time points in response to BR and auxin treatments (data not shown).

### Shoot growth and gene expression levels in different grafting combinations and branch-types

**Fig. 11:**
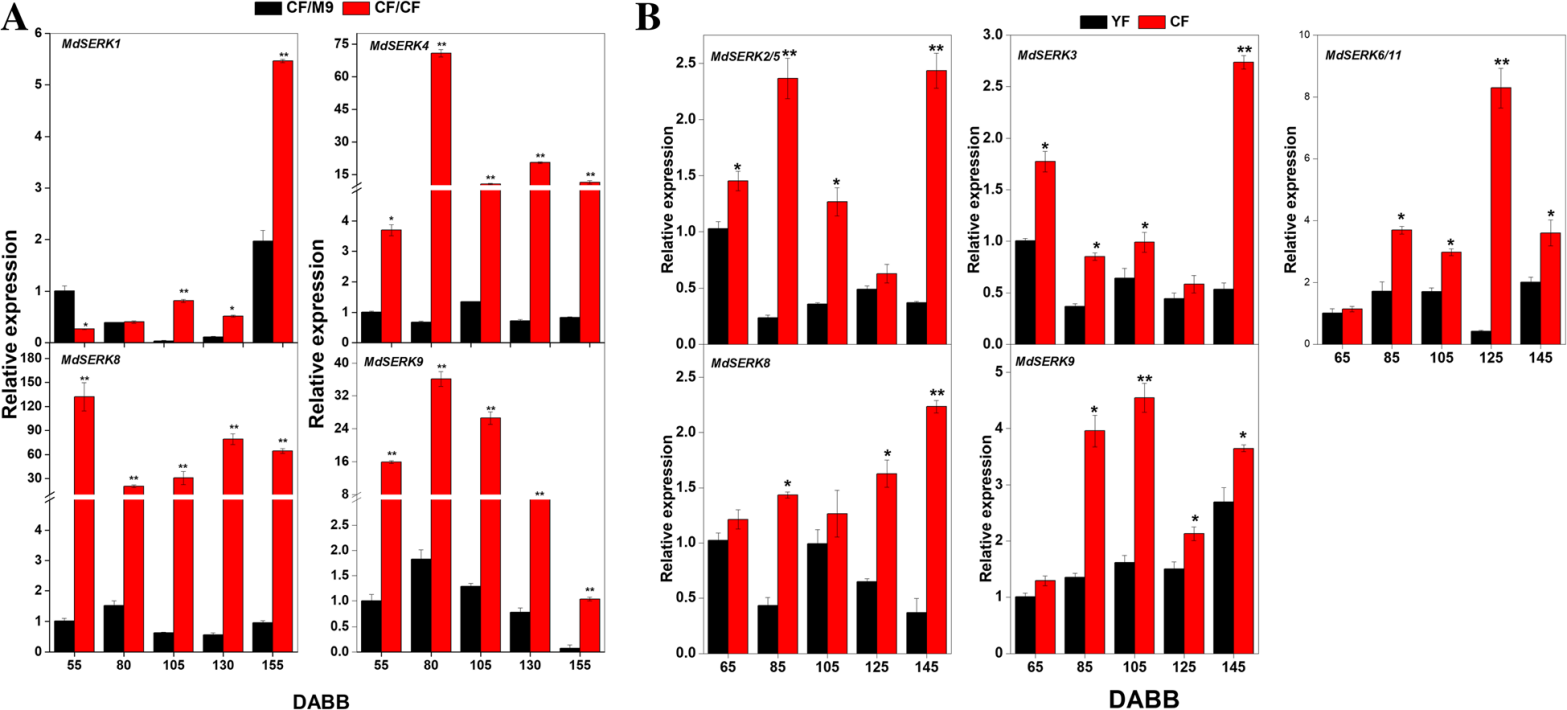
Comparison of *SERK* expression between grafting combinations (CF/CF vs. CF/M9) and cultivars (YF vs. CF). Quantitative reverse transcription polymerase chain reaction analysis of apple *SERK* genes in (**a**) CF/CF and CF/M9 after bud break (55, 80, 105, 130, and 155 days), and in (**b**) YF and CF after bud break (65, 85, 105, 125, and 145 days). *EF1-α* was used as an internal control. qRT-PCR data are shown relative to 55 days. Bars are mean ± standard error (*n* = 3). *Significant difference at 0.05 level, **significant difference at 0.01 level

The grafting combination of CF/CF (‘Nagafu No. 2’ (CF) on its own rootstock) grew faster than did CF/M.9 (CF on the dwarf rootstock M.9) after bud break, and the primary shoot length of CF/CF was greater than that of CF/M9 at 130 (by about 13 cm) and 155 (by about 42 cm) days after bud break (DABB) (Additional file 9: Figure S8). To investigate whether *MdSERK*s are involved in regulating apple tree height, *MdSERK* expression patterns were analyzed in the shoot tips of the two grafting combinations (Fig. 11a). *MdSERK1* showed higher expression levels in CF/CF trees than in CF/M.9 trees at 105, 130, and 155 DABB, but was repressed at 55 DABB in CF/CF trees. *MdSERK4* and *MdSERK8–9* were expressed at higher levels in CF/CF trees than in CF/M.9 trees from 55 to 155 DABB. *MdSERK4* expression was 3- to 75-fold higher in CF/CF trees than in CF/M.9 trees from 55 to 155 DABB. *MdSERK8* and *MdSERK9* also showed higher expression levels (by about 20- to 145-fold) in CF/CF trees than in CF/M.9 trees throughout the trial period. The expression patterns of the remaining *MdSERK* genes were irregular after bud break (data not shown).

A previous study showed that shoot length and internode length are shorter in YF (the spur-type bud mutation of “Yanfu 6”) than in CF, and that YF stops growing earlier than CF [38]. We also investigated *MdSERK* expression patterns in YF and CF shoot tips during the shoot growth period (Fig. 11b). Seven *MdSERK* genes showed high expression levels in CF. *MdSERK2/5* and *MdSERK3* were induced at 65, 85, 105, and 145 DABB in the shoot tips of CF. In CF, the expression levels of *MdSERK6/11* and *MdSERK9* were high from 85 to 145 DABB. *MdSERK9* expression was upregulated by about 2-, 1.5-, and 5-fold at 85, 125, and 145 DABB in standard-type CF. The expression of other *MdSERK* genes showed no particular pattern in YF and CF, like in CF/CF and CF/M.9.

## Discussion

*SERKs* have been shown to play important roles in the response to stress signals and in regulating plant growth, but they have only been systematically identified in model plants so far [1–4, 39]. The functional identification of all members in this family in apple, a woody fruit tree, had not yet been reported. Therefore, in this study, we conducted the first genome-wide identification and functional prediction of *SERKs* in apple. These results provide insights for further functional identification of *SERK* genes.

### Genome-wide identification and characteristics analysis of *SERK* genes in apple

Twelve apple SERK proteins were identified (Fig. 1; Table 1). All MdSERK proteins contained conserved SERK domains: the SP domain, the ZIP domain, five LRR domains, an SPP domain, a TM domain, a cytoplasmic Ser/Thr kinase domain, and the CTD (Fig. 2; Additional file 2: Figure S1). Structural analyses of the MdSERK proteins revealed conservation in the proportion of α helices, β sheets, extended strands, and random coils (Additional file 3: Figure S2; Additional file 1: Table S6). Protein structure determines its function, and these observations suggested that the MdSERK proteins have similar secondary structures and functions. Several characteristics, including protein length, molecular weight, instability index, GRAVY value, major aa content, and aliphatic index were compared (Table 1 and Additional file 1: Table S4). Most of the MdSERK proteins showed similar length and molecular weight to those of AtSERKs, indicating that MdSERKs are high molecular weight proteins [39]. Almost all of MdSERK proteins were predicted to be stable according to their low instability index (< 40). All MdSERK proteins had very similar GRAVY values and high aliphatic indexes, which may be associated with the contents of major hydrophilic and aliphatic amino acids (i.e., Leu, Ser, Gly, Ala, and Val). Most MdSERK proteins contained at least one transmembrane segment (Additional file 4: Figure S3), and all of them were predicted to locate in the cell membrane (Additional file 1: Table S4), consistent with their predicted functions as cell membrane receptors [29].

### Evolutionary and syntenic relationships of *SERK* genes

In the unrooted phylogenetic tree, the putative apple SERK proteins were grouped into different classes (Fig. 3). The 12 apple *SERK* genes were distributed into three groups, which may have different roles. Group A contained eight apple *SERK*s (*MdSERK2–3, MdSERK5–7*, and *MdSERK10–12).* The proteins encoded by these genes may play roles in plant immunity, stress resistance, and organ development, based on their similarities to OsSERK1–2, TaSERKlike3, and PoapSERKlike2 [6, 20, 40]. *MdSERK1* and *MdSERK8–9* were found to have close relationship with *AtSERK3–4*, *AtSERK5-Ler, MtSERK1–6, GmSERK2*, *SISERK3A/B,* and *NbSERK3A/B* in Group B. The proteins encoded by these genes are likely to be involved in BR-mediated biological processes, including stem growth, leaf development, root formation, and cell division, as well as in biotic and abiotic stress responses [4, 29]*. MdSERK4* shared high homology with *AtSERK1–2* and *MtSERK2,* which play roles in embryo development and disease resistance [6, 18]. There were several sister pairs in the combined tree, including four apple/apple pairs. The coding sequences and gene structures of the apple sister pairs were highly conserved (Fig. 3; Additional file 2: Figure S1; Additional file 5: Figure S4). All the MdSERKs were similar in their conserved domains, including the SP domain, the ZIP domain, five LRR domains, the SPP domain, the TM domain, the cytoplasmic Ser/Thr kinase domain, and the CTD. They all contained an intact RD motif, which is essential for SERK protein function [28]. These findings suggested that the functions of these apple *SERK* genes may be various and redundant.

Our results showed that there are more *SERK* genes in apple than in *A. thaliana* [39]. Gene duplication events may have played a significant role in the expansion of the apple SERK gene family. Tandem duplications and segmental duplications are the major evolutionary patterns [40]. Some gene duplication events for SPL, CYS, and bZIP genes have been characterized [41–43]. One tandem and five segmental duplications were found among the *MdSERK* genes (Fig. 4). Apple has undergone a genome-wide duplication that led to the formation of the present 17 chromosomes from an initial nine ancestral chromosomes. This event may have played an important role in the expansion of *MdSERK* genes [44], and may explain why there are more *MdSERK* genes than *AtSERK* genes.

Gene function in less-studied plants can be better understood through genomic comparison with a well-identified species, such as *Arabidopsis.* In our study, six *SERK* orthologs were identified from apple and *Arabidopsis* (Fig. 5; Additional file 1: Table S8)*.* Both *AtSERK4* and *AtSERK5-Ler* are involved in the response to cell death and stem elongation [4, 22, 29]. *MdSERK1* showed strong similarity to these genes, suggesting that it may have similar roles. *MdSERK3* was identified as a homolog of *AtSERK1* and *AtSERK2,* which play important roles in reproductive growth [18, 19]. *AtSERK3* (also known as *AtBAK1*) is involved in BR signal transduction, plant growth, and stress responses [22, 32]. *MdSERK8* and *MdSERK9* were identified as homologs of *AtSERK3,* indicating they may have diverse functions. The functions of these *MdSERK* genes should be confirmed experimentally in further studies.

### Expression and potential functional analysis of *MdSERK* genes

The 12 analyzed *MdSERK* genes were expressed differently among the roots, xylem, and phloem of stems, and the leaves and shoot tips of *M. hupehensis* seedlings (Fig. 6), consistent with *SERK* expression patterns in other species [8, 41, 42]. Almost all of the analyzed *MdSERK* genes showed higher expression levels in the xylem, phloem, or shoot tips than in other organs. Therefore, these *SERKs* may play critical roles in stem development. We also analyzed the expression of *MdSERK* genes in different tissues based on ArrayExpress data (Additional file 8: Figure S7). The highest expression levels of *MdSERK* genes were in the flowers, fruit, and leaves, indicating that they may play important roles in flower, fruit, and leaf development. The various expression patterns might be related to gene chromosomal locations, characteristics, and structures. The experimental and digital investigation data indicated that *MdSERK* genes are involved in many aspects of apple development. These findings should be confirmed by further functional analyses.

Several studies have reported that *SERK* genes are involved in defense signals and are master regulators of abiotic and biotic stresses in many plant species [6, 20, 43, 44]. *OsSERK1* is associated with host cell death, blast fungus, and defense signaling molecules [6]. The regulation of the rice immune receptors XA21 and XA3 by *OsSERK2* confers immunity to bacterial leaf blight [20]. Salinity and powdery mildew induce *SERKs* in barley [45]. *AtSERK3/AtBAK1* interact with flagellin-sensitive 2 (FLS2) and elongation factor Tu receptor, and are involved in flagellin sensing and binding of a bacterial elicitor, respectively [46, 47]. Plant cell death was shown to be controlled by *AtSERK3/AtBAK1* and *AtSERK4/AtBKK1* [21]. Previous studies have confirmed that SA, MeJA, and ABA have important roles in plant resistance [48–50]. Plants can upregulate many resistance genes, such as those encoding WRKY, YTH domain-containing RNA-binding proteins (YTPs), and cystatins (CYS), to adapt to salt stress [48, 50]. However, little is known about the potential roles of *SERK* genes in regulating stress responses in apple, or whether apple *SERK* genes are associated with MeJA, SA, ABA, and salt signals. Therefore, we monitored the expression patterns of apple *SERK* genes under various stress conditions. Some *MdSERK* genes were induced by several signals, including MeJA, SA, ABA, and salt stress (Fig. 7). For example, MeJA induced the expression of *MdSERK2/5, MdSERK3, MdSERK6/11,* and *MdSERK12*; SA treatment increased the expression levels of *MdSERK2/5* and *MdSERK12*; ABA treatment increased the transcript levels of *MdSERK2/5, MdSERK3,* and *MdSERK6/11*; and salt treatment upregulated *MdSERK4*, *MdSERK6/11,* and *MdSERK10.* Stress-specific elements (MBSs, TCA elements, CGTCA motifs, and ABREs) may partly explain the responses of some *MdSERKs* to MeJA, SA, and ABA signals (Additional file 6: Figure S5). These results suggested that these *MdSERK* genes may play important roles in plant responses to various stresses (SA, MeJA, ABA, and salt) through complex regulatory mechanisms.

Previous studies have concluded that the *SERKs*-mediated BR signaling is important for regulating plant growth. Previous studies have shown that *serk* mutants display abnormal growth, including dwarfism, delayed growth, stamen abortion, and a narrow-leaf phenotype [24–26]. Auxin was the first plant hormone shown to play important roles in many aspects of plant growth, including cell differentiation [51], plant structure [52], and apical meristem dominance [53]. Early research showed that auxin is involved in BR synthesis and signal transduction in model plants [54], and participates in the regulation of BR-responsive gene expression [55–58]. However, the relationship between *SERK* genes and BR or auxin signaling pathways in apple has not been explored. In this study, *MdSERK1–5* and *MdSERK8–9* in shoot tips were induced by BR, and the expression levels of *MdSERK1* and *MdSERK8–9* were increased by an auxin treatment (Fig. 10). The application of exogenous BR and auxin in separate experiments increased endogenous BR and IAA contents (Fig. 8), and induced horizontal and vertical growth of the stem (Fig. 9). These results indicate that there may be a complicated mechanism by which auxin regulates *SERK* genes in apple, and that these *MdSERK* genes might have a positive role in regulating apple tree growth by mediating BR and auxin signals.

Previous studies have shown that dwarf rootstocks can inhibit apple tree growth [52, 59, 60]. *SERK3–5* encode important regulators of plant growth [24–26]. However, it was unknown whether *MdSERK* genes participate in controlling apple tree growth on dwarf rootstocks. In this study, the transcript levels of *MdSERK1, MdSERK4,* and *MdSERK8–9* (homologs of the important growth regulators *AtSERK4/5* and *AtSERK3*) (Fig. 3) varied considerably among trees with different vigor (Fig. 11a and Additional file 7: Figure S6). These observations were consistent with their expression patterns in response to exogenous auxin treatment (Fig. 10). Compared with CF/CF, the dwarf phenotype of CF/M.9 apple trees may be caused by a lowered auxin content, according to previous studies [52, 60–62]. There was at least one TGA element (auxin-responsive element) in the promoters of *MdSERK1* and *MdSERK9* (Additional file 6: Figure S5)*.* These results indicated that *MdSERK1* and *MdSERK8–9* may be involved in the regulation of the dwarf phenotype of CF/M.9 trees through auxin signaling, and that *MdSERK4* may also play an important role in apple tree growth. However, few studies have focused on the roles of SERK proteins in the dwarfing mechanism of apple rootstocks. Our study has highlighted potential roles of *MdSERK* genes in the dwarfism of apple trees. Further research should explore the potential relationships between MdSERK proteins and dwarfism.

The *MdSERK* genes exhibited different expression patterns between CF and YF apple cultivars, which differ in their shoot elongation [38]. *MdSERK2–3, MdSERK5–6, MdSERK8–9,* and *MdSERK11* showed higher expression levels in CF than in YF during the shoot growth period (Fig. 11b), suggesting that they may be responsible for the growth difference between YF and CF. This expression pattern might be related to GA and auxin levels, as these hormones control the growth of YF trees [38]. At least one GARE or auxin-response motif was identified in the promoters of *MdSERK2–3, MdSERK5–6, MdSERK8–9,* and *MdSERK11* (Additional file 6: Figure S5), and close relationships between BR and GA or auxin have been identified in other plants [30, 36, 63]. The synteny analysis showed that *MdSERK8/9* and *AtSERK3* (an important regulator of shoot growth) may be heterogenous homologs (Fig. 5). These results indicate that *MdSERK* genes may be involved in the response to GA and auxin signals to regulate the growth of YF trees. This hypothesis needs to be confirmed experimentally in further studies.

## Conclusion

This study represents the first genome-wide analysis of the apple *SERK* family. Detailed bioinformatics analyses including gene chromosomal location, multiple sequence alignment, phylogenetic, synteny, chemical characterization, gene structure, promoter sequence, and protein–protein interaction analyses were conducted. The expression patterns of *MdSERK*s in different tissues, in response to various stresses and hormone treatments, and in different grafting combinations and apple cultivars were analyzed. Eight *MdSERK* genes (*MdSERK2–6* and *MdSERK10–12*), especially *MdSERK2* and *MdSERK5,* were predicted to play important roles in stress responses. Like *SERK3–5* in *Arabidopsis*, most *MdSERK* genes (*MdSERK1–6*, *MdSERK8–9,* and *MdSERK11*), especially *MdSERK8* and *MdSERK9,* appeared to be involved in regulating plant size. The data generated in this study provide information about the activities of apple *SERK* genes and help to support hypotheses on the involvement of *SERK* genes in stress responses and growth regulation. Future research should focus on validating the functions of *MdSERK* genes in various stress responses, and their roles in controlling apple tree size.

## Methods

### Chromosomal location, multiple sequence alignment, protein structure, chemical characterization, subcellular localization, phylogenetic, and intron/exon structure analyses of apple *SERK* genes

Known AtSERK (AtSERK1–4 and AtSERK5-Ler) protein sequences [7] were used as queries to search the Apple Genome Database (GDR) (https://www.rosaceae.org/). Pfam (http://pfam.xfam.org/) and SMART (http://smart.embl-heidelberg.de/) were used to confirm the presence of conserved pfam codes and SERK domains. Apple sequences lacking the characteristic domains present in AtSERK protein sequences were eliminated from further analyses.

Details of the chromosomal locations of *MdSERK* genes were obtained through BLASTN searches of the GDR database (GDR; http://www.rosaceae.org/). The *MdSERK* genes were mapped to chromosomes using MapDraw [64]. A BLASTN search of apple expressed sequence tag (EST) datasets (https://www.rosaceae.org/tools/ncbi_blast) from the Apple Genome Database was conducted to find the corresponding record for each putative *SERK* gene.

Multiple aa sequences were aligned using DNAMAN (version 6.0). The MEME program (http://meme-suite.org/) was used to identify conserved motifs, with the following parameters: number of repetitions-any; maximum number of motifs-10; optimum motif width-6 to 200 amino acid residues. Transmembrane helices were analyzed using the TMHMM server v. 2.0 (http://www.cbs.dtu.dk/services/TMHMM/) and the PHYRE server v. 2.0 (http://www.sbg.bio.ic.ac.uk/phyre2/html/page.cgi?id=index). MdSERK protein three-dimensional structures were predicted using NPS (https://npsa-prabi.ibcp.fr/cgi-bin/npsa_automat.pl?page=npsa_sopma.html). The ExPASy program (http://web.expasy.org/protparam/) was used to predict MdSERK protein characteristics. The subcellular localization of MdSERKs was predicted using Plant-mPLoc (http:// www.csbio.sjtu.edu.cn/bioinf/plant-multi/). Phylogenetic trees were prepared with MEGA7 using the maximum likelihood method, complete deletion, and bootstrap tests with 1000 replications. The intron/exon structures of apple *SERK* genes were analyzed using the Gene Structure Display Server (http://gsds.cbi.pku.edu.cn/).

### Synteny analysis, identification of *cis*-acting elements in promoters of apple *SERK* genes, and SERK protein interaction networks

Tandem and segmental duplications of apple genes can produce homologous genes. The *SERK* genes were considered as duplicates if the aligned gene sequences were at least 70% identical, and the length of the matching sequences corresponded to at least 70% of the longer gene [65, 66]. Duplicated genes separated by five or fewer genes within a 100-kb region on the same chromosome were considered as tandem duplicates [34]. Coparalogs located on duplicated blocks of different chromosomes were considered to be segmental duplications, as proposed by other researchers [38]. Syntenic blocks in apple as well as those between the apple and *A. thaliana* genomes were downloaded from the plant Genome Duplication Database (http://chibba.agtec.uga.edu/duplication) [39, 67] to identify segmentally duplicated *MdSERK* genes, and apple and *A. thaliana SERK* homologs. Circos (version 0.63) (http://circos.ca/) was used to visualize the chromosomal locations of *SERK* genes.

The upstream regions (− 1.5 kb upstream of the transcription start site) of apple *SERK* genes were used as queries to search the MEME (http://meme-suite.org/) and PlantCARE (http://bioinformatics.psb.ugent.be/webtools/plantcare/html/) databases to identify putative *cis*-elements.

*Arabidopsis* is the most popular model plant species and the functions of *AtSERK* genes have been well characterized. The role of similar genes in other species can be predicted based on their homologs in *Arabidopsis* [50]. Therefore, to further analyze the roles of apple *SERK* genes and the relationship between them and other genes, the interolog proteins from *Arabidopsis* were used to construct an interaction network using STRING 10 (http://string-db.org/) (option value > 0.800).

### Plant materials and treatments

The following six plant materials were used for four experimental treatments in this study: ‘NagafuNo. 2’ (CF)/ Malling 26 (M.26), *M. hupehensis* seedlings, CF/Malling 9 (M.9)-CF/CF, and CF/M.26-‘Yanfu 6’ (YF)/M.26. These materials were subjected to stress treatments, BR and auxin treatments, and used for grafting combination and branch-type comparisons, as described below.

*Stress treatment* For the stress experiments, scion buds of CF were grafted onto 1-year-old M.26 rootstock in 2014. After grafting, trees were grown in pots containing culture medium (mixture of garden soil and river sand at a 1: 1 ratio) and were drip-irrigated in a net house. In 2016, young leaves (third to fifth fully expanded leaves beneath the shoot apex) of CF/M.26 were treated with 50 μM MeJA, 100 μM SA, or 300 μM ABA at 55 DABB. The MeJA, SA, and ABA were purchased from Sigma (Deisenhofen, Germany) and were dissolved in ethanol. Leaves sprayed with sterile distilled water served as controls. Young leaves were harvested from both the control and treated lines at 0, 1, 3, 6, and 12 h after treatments [68]. The salt treatment was performed by irrigating plants with water containing 200 mM NaCl (dissolved in sterile distilled water). Trees irrigated with the same volume of sterile distilled water served as controls. Young leaves from salt-treated and control trees were collected at 0, 2, 4, 6, and 8 d after treatment [69].

*Hormone treatment* On March 1 in 2015 and 2016, two batches of *M. hupehensis* seeds were respectively planted in an experimental orchard at the Northwest Agriculture and Forestry University in Yangling (108°04′E, 34°16′N), China. When the two batches of seedlings reached the 8–10 leaf stage, they were transplanted into ½-strength Hoagland’s nutrient solution, and grown under greenhouse conditions (24 h cycle of 12-h light (800 μmol m^− 2^ s^− 1^) at 25 ± 1 °C, followed by 12-h dark at 15 ± 1 °C) on April 1 in 2015 and 2016. Equal-sized *M. hupehensis* seedlings, which were apomictic and very uniform in their genotype and phenotype [30, 70, 71], were used to assess the effects of BR (in 2015) and auxin (in 2016) treatments, and were evaluated at 2 months after the treatments. Using a hydroponic system, BR and auxin experiments were performed in greenhouse from June 1 to June 28 in 2015 and 2016, respectively. For the BR treatment, equal-sized seedlings (about 9.5 cm height) were treated with 0.5 mg/L 2,4-epibrassinolide (a BR) in aerated ½-strength Hoagland’s nutrient solution from June 1 to June 28, 2015. The optimum concentration of BR was determined based on previous studies [30, 72]. The BR was purchased from Sigma and was dissolved in ethanol. From June 1 to June 28 of 2016, the second batch of *M. hupehensis* seedlings (about 9 cm height) was cultured in aerated ½-strength Hoagland’s nutrient solution containing 0.1 mg/L 1-naphthylacetic acid (NAA, an auxin analogue, Sigma), which had been dissolved in ethanol. The optimal concentration of NAA was determined based on previous studies [73, 74]. Seedlings cultured in aerated ½-strength Hoagland’s nutrient solution and treated with the same volume of distilled water served as controls in 2015 and 2016. During the experimental process in both years, plants were grown in ½-strength Hoagland’s nutrient solution in black open-topped plastic containers (50 cm × 35 cm × 15 cm) and the solution was replaced every week. The seedlings were positioned at the top of the containers using a polystyrene foam board with holes. The seedlings were held firmly in the holes by sponges. There were 30 seedlings per container, and they were aerated by an air pump. Roots (consisting of the new root and root tips), xylem and phloem of stems (near the apices of newly growing shoots and 3–4 mm in diameter), leaves (third to fifth fully expanded young leaves beneath the shoot apices), and shoot tips (shoot apices) were collected at 0, 1, 2, 3, and 4 weeks after the hormone treatments. Roots were cleaned with distilled water and then excess liquid was removed with paper towels.

*Comparison of grafting combinations* We compared CF on its own roots with CF on the dwarf rootstock M.9 (CF/CF vs. CF/M.9). The scion buds from CF were grafted onto 1-year old CF (vigorous rootstock) and M.9 (dwarf rootstock) in 2012 and then planted in a net house in an experimental plot at the Yangling National Apple Improvement Center, Yangling, China (34.31°N, 108.04°E). The growth conditions of the trees were the same as those described in the ‘*Stress treatment*’ section. In 2013, the shoot tips (shoot apices) of CF/CF and CF/M.9 were collected for analysis at 55, 80, 105, 130, and 155 DABB.

*Branch-type comparisons* In 2009, standard-type CF and the short-branched spur-type mutant YF were each grafted onto Malling 26 (M.26) using standard horticultural practices. The grafted plants were grown in an experimental plot at the Yangling National Apple Improvement Center, Yangling, China (34.31°N, 108.04°E). In 2013, branch tips (branch apices) were sampled for RT-qPCR analysis of *MdSERK* gene expression at 65, 85, 105, 125, and 145 DABB.

To minimize sampling error, all samples were sampled using three biological replicates with six trees per replicate. All samples were collected in the same manner, and were cut into pieces and immediately placed in an ultra-low temperature freezer.

### Measurements of tree growth and endogenous hormones in *M. hupehensis* seedlings

*Hormone treatments* The plant height of *M. hupehensis* seedlings was measured at 0, 1, 2, 3, and 4 weeks after BR or auxin treatment in 2015 and 2016. Using a steel tape measure, the plant height was measured from the basal stem to the primary shoot apex. Stem diameter was the average value of the stem diameter measured in two directions with a Vernier caliper. Using ELISA, the endogenous BR and IAA contents in shoot tips were measure after the BR treatment (2015) and the auxin treatment (2016) as described elsewhere [75, 76].

*Comparison of grafting combinations* The primary shoot length (from the graft union to the primary shoot apex) of CF/CF and CF/M.9 was measured at 55, 80, 105, 130, and 155 DABB with a steel tape measure.

*Branch-type comparison* The growth characteristics of YF and CF were investigated and have been published elsewhere [38].

The above data were measured with three biological replicates with six trees per replicate to minimize experimental error.

### Apple *SERK* gene expression analysis

Total RNA was extracted using the CTAB method [77]. RNase-free DNase I (Invitrogen, Shanghai, China) was used to remove any residual genomic DNA. First-strand cDNA was synthesized from 2 μg total RNA using the SYBR Prime Script RT-PCR Kit II (TaKaRa, Dalian, China). The cDNA samples were diluted to about 150 ng/μL, and 1 μL (in a final volume of 10 μL) was used for qRT-PCR assays, which were conducted using three technical replicates and three biological replicates. Primers specific for *MdSERK* genes were designed using Primer 6 and Primer 3 (Additional file 1: Table S1). The specificity of primers was checked in GDR (https://www.rosaceae.org/). The coding sequences of *MdSERK2* and *MdSERK5*, and of *MdSERK6* and *MdSERK11* were extremely similar, so it was impossible to separate them by PCR using different pairs of primers. Therefore, only two pairs of primers were designed to amplify these genes. The qRT-PCR analyses were conducted using the SYBR Green qPCR kit (TaKaRa) and the Bio-Rad CFX 134 Connect Real-Time PCR Detection System (Bio-Rad, Hercules, CA, USA. The PCR amplification conditions were as follows: 95 °C for 5 min; 40 cycles of 94 °C for 20 s, 59 °C for 20 s, and 72 °C for 10 s. The apple *EF1-α* gene [27, 68] served as the internal standard. Relative gene expression levels were calculated using the 2^−ΔΔCt^ method [78, 79].

The young leaves of CF/M.26 were used to extract RNA, which was used for functional analyses of *MdSERKs* in response to stresses. The shoot tips of *M. hupehensis* seedlings, CF/CF, CF/M.9, and CF-YF were used to analyze the roles of *MdSERKs* in apple tree growth. The shoot tips are essential for plant architecture [27, 38]. We extracted RNA from the roots, xylem, and phloem of stems, leaves, and shoot tips of *M. hupehensis* seedlings in the control group to investigate the tissue expression patterns of *MdSERK* genes. Tissue expression patterns among different apple varieties and tissues were also investigated using data downloaded from the online ArrayExpress database (https://www.ebi.ac.uk/arrayexpress/, 240E-GEOD-42873).

### Statistical analysis

Standard values and standard error of experimental data were calculated using Microsoft Excel 2010. Analyses of the significance of differences were conducted using the Data Processing System (DPS, v7.05; Zhejiang University, Hangzhou, China).

## Additional files

Additional file 1: **Table S1.** Primers and their sequences used for qRT-PCR analyses. ^a^ Internal control. **Table S2.** Best hits for putative apple SERK proteins in BLAST searches against apple EST^a^ assemblies. ^a^ Downloaded from NCBI database. **Table S3.** Sequences of MdSERK proteins. **Table S4.** Amino acid composition, physical and chemical characteristics, and subcellular localization of MdSERK proteins. ^a^Grand average of hydropathicity ^b^Three main amino acids in each protein (A, Ala; S, Ser; G, Gly; L, Leu; V, Val). **Table S5.** Motif sequences identified using MEME tools. Motif numbers correspond to motifs in Additional file 2: Figure S1. **Table S6.** Secondary structures of MdSERK proteins. **Table S7.** Synteny analysis of *MdSERK* genes. **Table S8.** Synteny analysis of *MdSERK* and *AtSERK* genes. (DOCX 37 kb)

Additional file 2: **Figure S1.** Distribution of conserved motifs among apple *SERK* family members. Ten putative motifs are indicated with numbers in colored boxes. Names of all members and combined *E*-values are provided on the left; motif sizes are indicated below. Please see **Table S5.** for details of motifs. (TIF 540 kb)

Additional file 3: **Figure S2.** Predicted dimensional structures of MdSERK proteins. (TIF 5808 kb)

Additional file 4: **Figure S3.** Transmembrane topology analysis of MdSERK proteins. Transmembrane helices of the MdSERK proteins were predicted with TMHMM server v2.0. Red peaks indicate predicted transmembrane helices. (TIF 163 kb)

Additional file 5: **Figure S4.** Intron/exon organization of apple *SERK* genes. Phylogenetic analysis and intron/exon organization of apple *SERK* genes. Numbers above or below branches indicate bootstrap values. Differently colored areas correspond to genes in each group. (TIF 417 kb)

Additional file 6: **Figure S5.** Promoter sequences of selected apple *SERK* genes. Promoter (1.5-kb) sequences of 12 *MdSERK* genes were analyzed with PlantCARE and MEME software. (TIF 6224 kb)

Additional file 7: **Figure S6.** Interaction network for proteins encoded by apple *SERK* genes according to *Arabidopsis thaliana* orthologs. Line thickness is related to combined score. Homologous genes in apple are indicated in red font in parentheses. (TIF 1893 kb)

Additional file 8: **Figure S7**. Heat map showing *MdSERK* gene transcript levels in different tissues. Relative transcript levels are based on ArrayExpress data. (TIF 5289 kb)

Additional file 9: **Figure S8.** Investigation of primary shoot length in different grafting combinations. Primary shoot length of CF after grafting on CF and M.9 rootstocks at 55, 80, 105, 130 and 155 DABB. (TIF 5663 kb)

## Abbreviations

100daa: 100 days after flower
ABA: abscisic acid
BR: brassinosteroid
bri1–5: BR insensitive1–5
CF: Nagafu 2
CTD: C-terminal domain
CYS: cystatins
DABB: days after bud break
DGE: digital gene expression
DPS: Data Processing System
EST: expressed sequence tag
GA: gibberellin
GDR: Apple Genome Database
IAA: Indole-3-acetic acid
LRRII-RLK: leucine-rich repeat II receptor-like kinase
LRRs: leu-rich repeat
M.9: Malling 9
Mb: megabases
MeJA: methyl jasmonate
NAA: 1-naphthylacetic acid
NIK1–3: NSP-interacting kinase1–3
qRT-PCR: quantitative reverse transcription polymerase chain reaction
SA: salicylic acid
SAC52: SUPPRESSOR OF ACAULIS 52
SERKs: Somatic embryogenesis receptor-like kinases
SP: signal peptide
SPP: serine-proline-rich
TM: transmembrane
YF: Yanfu 6
YTPs: YTH domain-containing RNA-binding proteins
ZIP: leu-zipper
