# Supplementary material for: Genome-wide identification of SERK genes in apple and analyses of their role in stress responses and growth
Source: BMC Genomics. 2018 Dec 27;19:962. doi: 10.1186/s12864-018-5342-1 (PMC6307271; doi:10.1186/s12864-018-5342-1)
Supplement: Supplementary file 1 — Table S1. Primers and their sequences used for qRT-PCR analyses. a Internal control. Table S2. Best hits for putative apple SERK proteins in BLAST searches against apple ESTa assemblies. a Downloaded from NCBI database. Table S3. Sequences of MdSERK proteins. Table S4. Amino acid composition, physical and chemical characteristics, and subcellular localization of MdSERK proteins. aGrand average of hydropathicity bThree main amino acids in each protein (A, Ala; S, Ser; G, Gly; L, Leu; V, Val). Table S5. Motif sequences identified using MEME tools. Motif numbers correspond to motifs in Additional file 2: Figure S1. Table S6. Secondary structures of MdSERK proteins. Table S7. Synteny analysis of MdSERK genes. Table S8. Synteny analysis of MdSERK and AtSERK genes. (DOCX 37 kb) [file 12864_2018_5342_MOESM1_ESM.docx]

| Gene name | Forward primer (5’-3’) | Reverse primer (5’-3’) |
| --- | --- | --- |
| *MdSERK1* | TCCAGCCTCTCCACCTCCTC | GCAGCAACCCCTCCAGCAAT |
| *MdSERK2/5* | AAGTGGTGAGGATGCTCGAA | TATCCAAGCGCTGAACATGC |
| *MdSERK3* | TGCATCACACCAACCGAAAG | TTGCAGCCTTCACATCCCTA |
| *MdSERK4* | CCTGGTGGGGAGTTGCAGTT | AGAGGTGGTTGGTTTGGCGG |
| *MdSERK6/11* | CGGAAGTGTAGCCTCTCGAT | ATCTCCAACAACTGCCTCGA |
| *MdSERK7* | TCGAACTTGTCACTGGTCAGCG | TGTCCACAATGTCGTCAAGCCT |
| *MdSERK8* | TCTGAGGTGGTTCGAATGCT | TTGGTCCAGACAGCTCCATT |
| *MdSERK9* | TCCTCCTGTTTCTCCGAACC | TCGTCGCCAATAAGCAAGTG |
| *MdSERK10* | CAAATGCTTGGGGTCAGCTT | CCAGAAACACAAGGCTGCTT |
| *MdSERK12* | GCATTTGGTCTAAGCCTCGG | CTTGCTGCTGAAATTGTGCG |
| *EF1-α* | ATTCAAGTATGCCTGGGTGC | CAGTCAGCCTGTGATGTTCC |

**Table S1**

**Table S2**

| Gene | GenBank Accession No. | Score | E-value | Gene | GenBank Accession No. | Score | E-value |
| --- | --- | --- | --- | --- | --- | --- | --- |
| MDP0000291093 | CN891664 | 1236 | 0.0 | MDP0000131814 | CN911451 | 270 | 8e-71 |
| MDP0000935390 | GO541626 | 888 | 0.0 | MDP0000287771 | EB112427 | 1060 | 0.0 |
| MDP0000031416 | CN881157 | 1178 | 0.0 | MDP0000309283 | CN918121 | 1029 | 0.0 |
| MDP0000432466 | EG631347 | 1232 | 0.0 | MDP0000887896 | DT001946 | 1292 | 0.0 |
| MDP0000211724 | CV880418 | 823 | 0.0 | MDP0000196862 | GO513496.1 | 813 | 0.0 |
| MDP0000252094 | GO513496 | 1082 | 0.0 | MDP0000202785 | CN881157 | 1356 | 0.0 |

**Table S3**

| Gene name | Protein Sequence |
| --- | --- |
| MdSERK1 | MDPTLMTSSASASAFWLILLFGFFHLQKLAANVEGDALNALKSNLADPNNVLQSWDPTLVNPCTWFHFNSLVIAKIALHE  ISNIFAPPSDLGNANLSGQLVAQLGVLSKLQYLELYSNNITGTIPPELGGLSNLVSLDLYLNNLHGNIPETLGXLAKLRF  LRLNNNTLAGNIPMTLTKIESLQVLDLSNNNLTGDIPVNGSFSLFTPISFNNNPLLKPLPASPPPPLSPPPSSPGTTATG  AIAGGVAAGAALLFAAPAIALAYWRRRKPQDHFFDVPAEEDPEVHLGQLKRFSLRELQVATDTFSNKNILGRGGFGKVYK  GRLTDGTLVAVKRLKEERTQGGELQFQTEVEMISMAVHRNLLRLRGFCMTPTERLLVYPYMANGSVASCLRDRPEXQAAL  DWPKRQCIALGSARGLAYLHDHCDPKIIHRDVKAANILLDEEFEAVVGDFGLAKLMDYKDTHVTTAVRGTIGHIAPEYLS  TGKSSEKTDVFGYGVMLLELITGQRAFDLARLANDDDVMLLDWVKGLLKDRRLETLVDADLNGNYVEDQVEQLIQVALLC  TQGTPGDRPKMSEVVRMLEGDGLAERWEEWQKEEVFRQDYNPIHHPNTNWIIDSTSHIPPDELSGPR |
| MdSERK2 | MLPLKLLIFFLSSCSLCLSYEPRNHEVEALISLRVGLNDPHGVLNNWDEDSVDPCSWAMITCSPDNLVIGLGAPSQSLSG  TLSGAFANLTNLRQVLLQNNNICGKLPSELGTLPKLQTLDLSNNRFSGLVPDSLAHLNTLQYLRLNNNSLSGPFPVSLAK  IPDLAFLDLSYNNLSGPIPKFPARTFNVVGNPLICASSSTEGCSGSATPVPLSLSLKTSPGKHNSKTVAIALGLSLSCVL  VIVLLLGILWHRKKQKTQSILNISDIQEEGIVSLGNLRSFTFKQLQLATDNFSSKHILGAGGFGNVYKGKLPDGTMVAVK  RLKDVTGTAGESQFRTELEMISLAVHRNLLRLIGFCATFSERLLVYPYMSNGSVAARLRGKPALDWNTRKRIAIGAARGL  LYLHEQCDPKIIHRDVKAANVLLDDYCEAIVGDFGLAKLLDHADSHVTTAVRGTVGHIAPEYLSTGQSSEKTDVFGFGIL  LIELITGMRALEFGKTVNQKGAILEWVKKIQQEKKVEVLVDRELGNNYDRIEVGEMLQVALLCTQYLPAHRPKMSEVVRM  LEGDGLAEKWAASHNQSNSSMDRFQSHNSNKSSSHTDGIHSKHDGNERDRGSMFSAWIDEDEDENSLDSYAMELSGPR |
| MdSERK3 | MGMGGTKAALCFLAFLWSFTCGNGLLSPKGVNFEVQALMGIKGSLVDPHGVLDNWDDDSVDPCSWTMVTCSPESLVIGLG  TPSQSLSGTLSPSIGNLTNLQIVLLQNNNITGAIPSEIEKLSKLHTLDISNNMFTGAIPSSLGHLRSLQYLRLNNNSLSG  AFPVSLANMSQLAFLDLSYNNLSGPVPRFAAKTFNIIGNPLICATGSEAECNGTTLLPMSMNLNTTQTAFPRGPKSHKIA  LAFGLSLGCLCLIVLGFGAVIWWRQRRNQQAFFGVKDRHHEEISLGNLKRFHFRELQIATHNFSSKNILGKGGFGHVYKG  TLQDGTFVAVKRLKDGSALGGEIQFQTEVEMISLAVHRNLLRLYGFCITPTERLXVYPYMSNGSVASRLKGKPVLDWGTR  KRIALGAGRGLLYLHEQCDPKIIHRDVKAANILLDDYCEAVVGDFGLAKLLDHQDSHVTTAVRGTVGHIAPEYLSTGQSS  EKTDVFGFGILLLELITGQRALEFGKAANQKGAILDWVKRIHHEKKLEMLVDKDLKTNYDRIELEEMVQVALLCTQYLPS  HRPKMSEVVRMLEGDGLVERWEASQKAESAKSKAPEFSSSDRYSDLTDDSSLLVQAMELSGPR |
| MdSERK4 | MERKVGNSVCLWFILXAHPLWMTMVLANMEGDALHTLRTNLEDPNNVLQSWDPTLVNPCTWFHVTCNNENSVIRVELYSN  NMSGPIPSELGNLTSLVSLDLYLNSFSGLIPGTLGRLSKLRFLFANNMDLCGPVTGRPXPGSPPFSPPPPFVPPPPISTP  GGNSATGAIAGGVAAGAALLFAAPAIAFAWWRRRKPQEFFFDVPAEEDPEVHLGQLKRFSLRELQVATDSFSNKNILGRG  GFGKVYKGRLADGSLVAVKRLKEERTPGGELQFQTEVEMISMAVHRNLLRLRGFCMTPTERLLVYPYMANGSVASCLRER  PPNQPPLDWPTRKRIALGSARGLSYLHDHCDPKIIHRDVKAANILLDEEFEAVVGDFGLAKLMDYKDTHVTTAVRGTIGH  IAPEYLSTGKSSEKTDVFGYGIMLLELITGQRAFDLARLANDDDVMLLDWVKGLLKEKKLEMLVDPDLQSNYVEAEVEQL  IQVALLCTQGSPMDRPKMSEVVRMLEGDGLAERWDEWQKVEVLRQEVELAPHPXSDWIVDSTENLHAVELSGPR |
| MdSERK5 | MEIWKGGKALCFVAFFCLWSCATGLLSPKGVNYEVQALVAIKGALEDPRGVLRNWDETSVDPCIWNMVTCSLDGLVIGLG  TPSQNLSGTLSPSIGNLTNLQLVTFQDNHITGTIPAELGRLQKLQTLDLSSNLLNGEIPSTLSHLKSLQYMDMSYNNLSG  PVPRFPARTFKFLNFXICFAQVXLLQIMIFMIQKLCCKNPNVLFLMCQDSQPAGRPRSHKIALAFASSLGCICLLILGFE  QHHEEVCLGNLRSFHFRELQSATHNFSSKNLVGKGGFGNVYKGCLRDGTVIAVKRLKDANAIGGEIQFQTELEMISLAVH  RNLLRLYGFCMTAKERLLVYPYMSNGSVASRLKAKPALDWNTRKRIALGAGRGLLYLHEQCDPKIIHRDVKAANILLDDY  YEAVVGDFGLAKLLDHHDSHITTAVRGTVGHIAPEYLSTGQSSEKTDVFGFGILILELISGQRALEFGKAANQKGAILDW  VKKIQQEKKFDVLVDKELKNDYDAIELEEMIQVALLCTQNLPNQRPKMSEVVRMLEGDGLAEKWEASQRAESNRCRANEF  SSSERYSDLTDDSSLLAQAMELSGPR |
| MdSERK6 | MGRSTFTLWKLGLLVLALVEAASATLSPTGVNYEVEALAAIKSDLRDPHNVLENWDSNSVDPCSWRMVTCTPDGYVSALG  LPSQSLSGILSPAIGNLSNLQSVLLQNNEISGPIPASIGNLEKLQTLDLSNNNFNGDIPDSLGNLKNLNYLIVGNPLICG  VKSENCSAVFPEPLSFPPDALKESDSGTKRHHMTIVLGASFSAAFGVIIIIGLLVWLRYRHNQQIFFDVNADQYDPEVCL  GHLKRYTFKELWAATDHFNSKNILGRGGFGIVYKGSLSDGTLVAVKRLKDYSTTGGEIQFQTEVEMISLAIHRNLLRLCG  FCSTENERLLVYPFMPNGSVASRLRDHIHGRPALDWARRKRIALGTARGLVYLHEQCDPRIIHRDVKAANILLDEDFEAV  VGDFGLAKLLDHRESHVTTAVRGTVGHIAPEYLSTGQSSDKTDVFGFGILLLELITGQKALDFGRVANQKGVMLDWVKKL  HQEGKLNLMVDKDLKGNIDRVELEEMVQVALLCTQFNPLYRPKMSEVLKMLEGDGLAEKWEASQKVETPRFRSCEHPRQR  YSDFIEESSLVVEAMELSGPRITLDWYESWSIAMMQSMQTKLVSVFRVQRAPATWKTSQSPTHSFPQTPPPRVTIPTDGV  NGNRYQCSMGLRTPPRVLDVIPRFVLLISSPDHANEIHISLAFAVDPLHCLSAMVWKKPAFPEQFYSPKRKVISGFGLGI  GVSVIVLGVLFFTDFFKTPAVETSIQGFYSLNSSSVSWPFSFSSSSETQDFVDKTHEANESIVAGKQNFTDLEKPHLGNF  TEEGKDGSFGVGGEMGKQKNSEKGSAIAENGNFLNSNGGGTSLEIAHLGNSSEVSKNGSLHGEEGRVNGNLSLSGKEDMH  AEKAVEGSFSRNTSSVDGNAAKIVKKKRAWKAAHRGNSTVKIADYDSQMGKMQTDLYQKCDIFNGRWVRDDSKPYYPGGS  CPYIDRDFNCHLNGRADSAFIKWKWQPNECDIPSLNATDFLMRLRGKRLVFVGDSLNRNMWESLDYNCSVDFVVSPFLVR  ESSFTSKNGTFETLRLDLMDRTTSMYHDADVIVFNTGHWWTHEKTSRGEDYYQEGNHVHPRLKVLEAYKRALTTWARWVD  KYIDVNRTQVFFRGYSVTHFSGGQWNSGGQCHKETEPIFNETYLANYPSKMRALEHVLKEMKSPVIYLNISRLTDYRKDG  HPSIYRMKYKTVEEQITAERSQDCSHWCLPGVPDAWNELLYAALLKAGWGSRKN |
| MdSERK7 | MSXVFSRSHPFKPIIKWLILLLLPTIGFASTEPDVEGEALTDLLTALKDSSGRITDWNDNFVSPCFSWSHVTCRNGNVIS  LDFQNNNLTGLLPVYLANLTHLQNLNLANNNFRGPIPNAWGQLSNLKHLDLSSNELTGRIPMQFFTIPNFNFTGSHLDCG  SSLKQPCVSGSSLQGLCFVQLYCFGLPIMFELPRNNXXLQNLAASNRKSKLGTVITSASCSVSVILLXGALFAYRYYRMH  KLKYDVFVDVAGEDECKISFGQLKRFSWREIQLATDNFDESNIIGQGGFGRVYKVKRLTDYNSPGGEAAFLREVQLISVA  VHRNLLRLIGYCTTPSERILVYPFMKNLSVAYRLRDLKPGEKGLDWSTRKHIAFGAAHGLEYLHEHCNPKIIHRDLKAAN  ILLDDKFEPVLGDFGLAKLVDTKSTHVTTQIRGTMGHIAPEYLSTGKSSEKTDVFGYGITLLELVTGQRAIDFARLEEEE  DVLLLDHIKKLLRENRLDDIVDRNMKMHDPKEVETVIQVALLCTQSSPEDRPKMAXVVRLLQGVDLAERWAEWEQLEGVR  NREFSLLSHQFAWAXESTHDQEAIQLSKAR |
| MdSERK8 | MTSSASVSVWLILXFGFFHLHKLAANVEGDALNALKTNLADPNNVLQSWDPTLVNPCTWFHVTCNSENSVTRVDLGNANL  SGQLVAQLGVLSKLQYLELYSNNITGTIPEELGGLADLVSLDLYLNKLHGTIPAALGXLAKLRFLRLNNNTLSXTIPLTL  TNIQSLQVLDLSNNXLTGDIPVNGSFSLFTPISFANNPLKPLPPSPPPPVSPNPPSSPGTTATGAIAGGVAAGAALLFAA  PAIALAYWRRRKPQDHFFDVPAEEDPEVHLGQLKRFSLRELQVATDTFSNKNILGRGGFGKVYKGRLADGTLVAVKRLKE  ERTQGGELQFQTEVEMISMAVHRNLLRLRGFCMTPTERLLVYPFMFNGSVASCLRDRPEGQAALDWPIRQRIALGSARGL  AYLHDHCDPKIIHRDVKAANILLDEEFEAVVGDFGLAKLMDYKDTHVTTAVRGTIGHIAPEYLSTGKSSEKTDVFGYGVM  LLELITGQRAFDLARLANDDDVMLLDWVKGLLKDRRLETLVDADLNGNYVDDQVEQLIQVALLCTQGTPGERPKMSEVVR  MLEGDGLAERWEEWQKEEVFRQDFNPIHHPRHXVNMKKKHCEPDEFTCTIMIRMVGKLGTGDECLGLFQEMVNKGCGPNM  MFYNTMIQALARSKMVDKAVVVFSKMVQNNCRPNEFTYSVVLNLLVAEGQLGGFDEVVGMSKKYMTKSIYAYLVRTLSKL  SHAGEAHRLFCNMWSFHDMGDRDALIVNRILSLYNSLINCLGKNGDVDEAHMRFKEMQEKGFSPDVVXYSTLIECXGKTD  RVEMACRLFDNMLAQGCYPNIVTYNILLDCLERYTXFCIIACLEPVSSSSQGRSMWLXLRLLKKLASHGAITTRTRFVRG  MSYLGMKIHAHSCEIEAAPLEVSTKNDSHLRNITNLVTDSQXLLKISIIDVEACLVFQIWCLTVFQN |
| MdSERK9 | MTSSASVSVWLILLFGFFHLHKLAANVEGDALNALKTNLADPNNVLQSWDPTLVNPCTWFHVTCNSENSVTRVDLGNANL  SGQLVAQLGVLSKLQYLELYSNNITGTIPEELGGLADLVSLDLYLNKLHGTIPAALGNLAKLRFLRLNNNTLSXTIPLTL  TNIQSLQVLDLSNNXLTGDIPVNGSFSLFTPISFANNPLKPLPPSPPPPVSPNPPSSPGTTATGAIAGGVAAGAALLFAA  PAIALAYWRRRKPQDHFFDVPAEEDPEVHLGQLKRFSLRELQVATDTFSNKNILGRGGFGKVYKGRLADGTLVAVKRLKE  ERTQGGELQFQTEVEMISMAVHRNLLRLRGFCMTPTERLLVYPFMFNGSVASCLRDRPEGQAALDWPIRQRIALGSARGL  AYLHDHCDPKIIHRDVKAANILLDEEFEAVVGDFGLAKLMDYKDTHVTTAGVMLLELITGQRAFDLARLANDDDVMLLDW  VKGLLKDRRLETLVDADLNGNYVDDQVEQLIQVALLCTQGTPGERPKMSEVVRMLEGDGLAERWEEWQKEEEMVNKGRAP  NMMFYSTMIQALARSKMVDKAVVVFSKMVQNNCRPNEFTYSVVLNLLVAEGQLGGFDEVVGMSKKYMTKSIYAYLVRTLS  KLSHALEAHRLFCNMWSFHDMGDRDAYTSMLECLCSAGKTAEAVDMLSNIHEKGITTDTIMYKVLAALGRLKQIPHLHEL  YERMKLDGPEPDIFTYNIMILSFGRAGKVHEAVTIFEHLENSDCKPDIISFTIR |
| MdSERK10 | MEIRTEGKALCFVAFFCFWTSAATGLLSPKGVNYEVQALVSIREALKDPRGVLRNWDGTSVDPCIWNXVTCSLDGLVIGL  GTPSQNLSGTLSPSIGNLTNLQLVTFQDNHITGSIPAELGRLQKLQTLDLSSNLFNGEIPSTLSHLKSLQYLRLNNNTLS  GAIPSSFANMTQLAFLDMSYNNLSGPVPRFPAKTFNVVGNPLICATGKEQDCSGTTRPPLSLPSNNSTNSQAAERPRSHK  IALAFASSLGCICLLILGFGFLLWWRQKHNKQIFLDVTEQHHEEVCLGNLRSFHFRELQAATHNFSSKNLVGKGGFGNVY  KGYLRDGTVIAVKRLKDTNAIGGEIQFQTELEMISLAVHRNLLRLYGFCMTAKERLLVYPYMSNGSVASRLKAKPPLDWS  TRKRIALGAGRGLLYLHEQCDPKIIHRDVKAANILLDDYCEAVVGDFGLAKLLDHHDSHITTAVRGTVGHIAPEYLSTGQ  SSEKTDVFGFGILILELISGQRALEFGKAANQKGAILDWVKKIQQEKKFDVLVGKELKNDYDPIEVEEMIQVALLCTQNL  PSQRPKMSEVVRMLEGDGLAEKWEASQRAESNRCRANEFSSSERYSDLTDDSSLLAQAMELSGPR |
| MdSERK11 | MKIEKDYMGRSNFMLCKLGLLVLALAEASSATLSPTGVNYEVEALAAIKSDLIDPHNVLENWDSNSVDPCSWRMVTCTPD  GYVSALGLPSQSLSGILSPAIGNLSNLQSVLLQNNAISGPIPTSIGNLVKLQTLDLSNNNFNGDIPDSLGNLKNLNYLDL  SFNNLSGSLPKISARTFKIVGNPLICGVKAANCSAVFPEPLSFPPDALKESDSGTKRRHMTIVLGASFSAVFGVIIIIGL  LVWLRYRHNQQIFFDVNADQYDPEVCLGHLRRYTFKELRAATDHFNSKNILGRGGFGIVYKGSLNDGTLVAVKRLKDYNT  AGGEIQFQTEVEMISLAVHRNLLRLCGFCSTENERLLVYPFMPNGSVASRLRDHIHGRPALDWARRKRIALGTARGLVYL  HEQCDPRIIHRDVKAANILLDEDFEAVVGDFGLAKLLDHRESHVSTAVRGTVGHIAPEYLSTGQSSDKTDVFGFGILLLE  LITGQRALDFGRVANQKGVMLDWVRSLSLSHACTHVKKLHQEGKLNLMVDKDLRGKLDRVELEEMVQVALLCTQFNPLYR  PKMSEVLKMLEGDGLAEKWEASQKVETPRQSWWLYFACSVLLPPARHHSRRLTASIRHHQASKSQRIGRVIGNHQCPHVM  LWASEQGHVSSYVSARNQIVFVSSRQLVQENSKFQKSSDKAAPPLRTINDAFIYAETLSQMTNMSTTYTIHPRPWFGRSS  FFSPRRKITSGFVLGIGVSVIVLGWLFFTNFLKSPAVETSIQGFDRLNSSSVPWPFSFSSSSEMQDFVDKTHEANESTVA  GKQNCTLLGEAHLGNFTEEVKDESFGAEGEMVLENGNLHGKEGRVIGNFSFSGKEDVHAEKAVDGSFSTNSSNVDGNVGK  FVMKKRAWKAAHRENSIAKIVEYDSQMVKMQTDLWVRDDSKPYYPGGSCPYIDRDFNCHLNGRPDNAFIKWKWQPNECDI  PSLNATDFLVRLRGKRMVFVGDSLNRNMWESLVCILRHSIRNKKRVHEISGKREFKKKGFYAFRFEASVIHLRMFLSEES  VFGMKISNHPWYSHTCRIIIVQWILLLLHSLLGNHHSRVKMTTSMYDDADVMVFNTGRWWTHEKTSRGEDYYQEGNYVHP  RLQVLEAYKRALTTWARWVDENIDVDRTQVFFRGYSVTHFSGGQWNSGGQCHKETEPIFNETYLGNYPSKMRALEHVIEE  MKTPVIYLNISRLTDYRKDGHPSIYRMKYKTVEERITAEGSQDCSHWCLPGVPDAWNELLYAALLKSGWGSRGN |
| MdSERK12 | MGIGIGMGMRGAKAALCFMAFLWSWTCGNGLLSPKGVNFEVQALMGLKNSLVDPHGVLDNWDDDSVDPCSWTMVTCSPES  LVIGLGTPSQSLSGTLSPSIGNLTNLQIVLLQNNNITGPIPREIERLSKLRTLDISNNFFTGAIPSSLGHLRSLQYFIVG  NPLICATGSEAQCNGTTLMPMLMNLTTTQAALPGRSKNHKVALAFGLSLGCLCLIVLGFGAVIWWRQRRNQQAFFDVKDR  HHEEISLGNLKRFHFRELQIATHNFSSKNILGKGGFGHVYKGTLQDGTFVAVKRLKDGSALGGEIQFQTEVEMISLAVHR  NLLRLYGFCITPAERLLVYPYMSNGSVASRLKGKPVLDWGTRKRIALGAGRGLLYLHEQCDPKIIHRDVKAANILLDDYC  EAVVGDFGLAKLLDHQDSHVTTAVRGTVGHIAPEYLSTGQSSEKTDVFGFGILLLELITGQRALEFGKAANQKGAILDWV  KKIHQEKTLEMLVDKDLKTNYDRIELEEMVQVALLCTQYLPGHRPKMSEVVRMLEGDGLVERWEASQKVESTKSKAPEFS  SSDRYSDLTDDSSLLVQAMELSGPR |

**Table S4**

| Proteins | Instability Index | GRAVY^a^ | Major Amino Acid^b^ | Aliphatic Index | PI |  | Localization |
| --- | --- | --- | --- | --- | --- | --- | --- |
| MdSERK1 | 41.30 | -0.134 | L(14.2%)A(8.1%)G(7.3%) | 97.56 | 5.45 |  | Cell membrane |
| MdSERK2 | 38.26 | -0.106 | L(14.1%)S(9.9%)G(7.8%) | 98.73 | 6.66 |  | Cell membrane |
| MdSERK3 | 39.19 | -0.073 | L(13.3%)G(9.1%)S(8.3%) | 94.70 | 7.60 |  | Cell membrane |
| MdSERK4 | 42.86 | -0.173 | L(12.5%)G(7.6%)V(7.4%) | 90.45 | 5.51 |  | Cell membrane |
| MdSERK5 | 40.90 | -0.067 | L(13.5%)G(7.7%)AS(7.2%) | 96.88 | 7.18 |  | Cell membrane |
| MdSERK6 | 35.25 | -0.335 | L(9.7%)S(8.9%)G(7.7%) | 81.61 | 6.70 |  | Cell membrane |
| MdSERK7 | 32.83 | -0.175 | L(13.2%)S(7.3%)G(6.4%) | 95.02 | 6.66 |  | Cell membrane |
| MdSERK8 | 39.17 | -0.061 | L(12.7%)V(7.2%)GA(7.0%) | 94.51 | 6.27 |  | Cell membrane |
| MdSERK9 | 35.37 | -0.067 | L(13.4%)A(8.1%)V(7.0%) | 96.90 | 5.86 |  | Cell membrane |
| MdSERK10 | 38.60 | -0.171 | L(13.0%)S(8.3%)G(7.8%) | 92.70 | 7.88 |  | Cell membrane |
| MdSERK11 | 39.08 | -0.300 | L(9.9%)S(8.9%)G(7.2%) | 84.38 | 8.69 |  | Cell membrane |
| MdSERK12 | 36.06 | -0.073 | L(13.2%)G(9.6%)S(7.4%) | 95.83 | 7.59 |  | Cell membrane |

**Table S5**

| Motif | Longth(aa) | Sequence |
| --- | --- | --- |
| 1 | 50 | GELQFQTEVEMISLAVHRNLLRLRGFCMTPTERLLVYPYMANGSVASRLR |
| 2 | 50 | YLHEQCDPKIIHRDVKAANILLDEEFEAVVGDFGLAKLLDHKDSHVTTAV |
| 3 | 50 | PEVHLGQLKRFSLRELQVATDNFSSKNILGRGGFGKVYKGRLADGTLVAV |
| 4 | 50 | RGTVGHIAPEYLSTGQSSEKTDVFGFGILLLELITGQRALDFGRLANQKG |
| 5 | 38 | GNYDRIEVEQLIQVALLCTQGSPGERPKMSEVVRMLEG |
| 6 | 62 | EGPEGLVIGLGLPSQNLSGTLSPSIGNLSNLQSLLLQNNNITGVLGSNLSGLSGNLLQLNIG |
| 7 | 29 | KSDLADPHNVLQNWDPTSVDPCTWFMVTC |
| 8 | 21 | GKPALDWPTRKRIALGAARGL |
| 9 | 21 | LDWVKGLLKEKRLEVLVDADL |
| 10 | 15 | DGLAERWEEWQKVEV |

**Table S6**

| Protein | α helix | | Extended strand | | Random coil | | β turn | |
| --- | --- | --- | --- | --- | --- | --- | --- | --- |
|  | Amino acid length | Proportion  (%) | Amino acid length | Proportion  (%) | Amino acid length | Proportion  (%) | Amino acid length | Proportion  (%) |
| MdSERK1 | 271 | 43.22% | 90 | 14.35% | 211 | 33.65% | 55 | 8.77% |
| MdSERK2 | 253 | 39.66% | 110 | 17.24% | 225 | 35.27% | 50 | 7.84% |
| MdSERK3 | 224 | 35.96% | 111 | 17.82% | 224 | 35.96% | 64 | 10.27% |
| MdSERK4 | 227 | 40.97% | 85 | 15.34% | 197 | 35.56% | 45 | 8.12% |
| MdSERK5 | 255 | 43.52% | 95 | 16.21% | 170 | 29.01% | 66 | 11.26% |
| MdSERK6 | 423 | 33.73% | 241 | 19.22% | 475 | 37.88% | 115 | 9.17% |
| MdSERK7 | 246 | 41.69% | 106 | 17.97% | 191 | 32.37% | 47 | 7.97% |
| MdSERK8 | 437 | 46.15% | 154 | 16.26% | 281 | 29.67% | 75 | 7.92% |
| MdSERK9 | 385 | 49.74% | 107 | 13.82% | 223 | 28.81% | 59 | 7.62% |
| MdSERK10 | 259 | 41.44% | 102 | 16.32% | 202 | 32.32% | 62 | 9.92% |
| MdSERK11 | 462 | 36.26% | 261 | 20.49% | 426 | 33.44% | 125 | 9.81% |
| MdSERK12 | 216 | 36.92% | 110 | 18.80% | 196 | 33.50% | 63 | 10.77% |

**Table S7**

| Region 1 (Apple) | | | Region 2 (Apple) | | | | Gene in the synteny region | | Gene name | |
| --- | --- | --- | --- | --- | --- | --- | --- | --- | --- | --- |
| Chromosome | Start | Stop | Chromosome | Start | Stop | Gene 1 | | Gene 2 | Gene 1 | Gene 2 |
| chr2 | 7587650 | 7694845 | chr15 | 42756445 | 43075246 | MDP0000291093 | | MDP0000287771 | MdSERK1 | MdSERK8 |
| chr2 | 7587650 | 7694845 | chr15 | 42756445 | 43075246 | MDP0000291093 | | MDP0000309283 | MdSERK1 | MdSERK9 |
| chr9 | 4209882 | 8287966 | chr17 | 4680317 | 8859450 | MDP0000031416 | | MDP0000202785 | MdSERK3 | MdSERK12 |
| chr13 | 2949565 | 8747953 | chr16 | 1636635 | 6821956 | MDP0000211724 | | MDP0000887896 | MdSERK5 | MdSERK10 |
| chr13 | 2949565 | 8747953 | chr16 | 1636635 | 6821956 | MDP0000252094 | | MDP0000196862 | MdSERK6 | MdSERK11 |

**Table S8**

| Region 1 (Apple) | | | Region 2 (*Arabidopsis*) | | | | Gene in the synteny region | | Gene name | |
| --- | --- | --- | --- | --- | --- | --- | --- | --- | --- | --- |
| Chromosome | Start | Stop | Chromosome | Start | Stop | Gene 1 | | Gene 2 | Gene 1 | Gene 2 |
| chr2 | 7157558 | 7804831 | Chr2 | 2140802 | 6826724 | MDP0000291093 | | AT2G13790 | MdSERK1 | AtSERK4 |
| chr2 | 7257351 | 7804831 | Chr2 | 2140802 | 6826724 | MDP0000291093 | | AT2G13800 | MdSERK1 | AtSERK5 |
| chr9 | 4100550 | 5263268 | chr1 | 26996809 | 27094027 | MDP0000031416 | | AT1G71830 | MdSERK3 | AtSERK1 |
| chr9 | 3832201 | 4968568 | chr1 | 12458670 | 12652646 | MDP0000031416 | | AT1G34210 | MdSERK3 | AtSERK2 |
| chr15 | 40256432 | 47075143 | Chr4 | 1118441 | 19136565 | MDP0000287771 | | AT4G33430 | MdSERK8 | AtSERK3 |
| chr15 | 39753425 | 45275206 | Chr4 | 1208001 | 18020597 | MDP0000309283 | | AT4G33430 | MdSERK9 | AtSERK3 |
