# Supplementary figures and images for: Genome-wide identification of SERK genes in apple and analyses of their role in stress responses and growth
Source: BMC Genomics. 2018 Dec 27;19:962. doi: 10.1186/s12864-018-5342-1 (PMC6307271; doi:10.1186/s12864-018-5342-1)

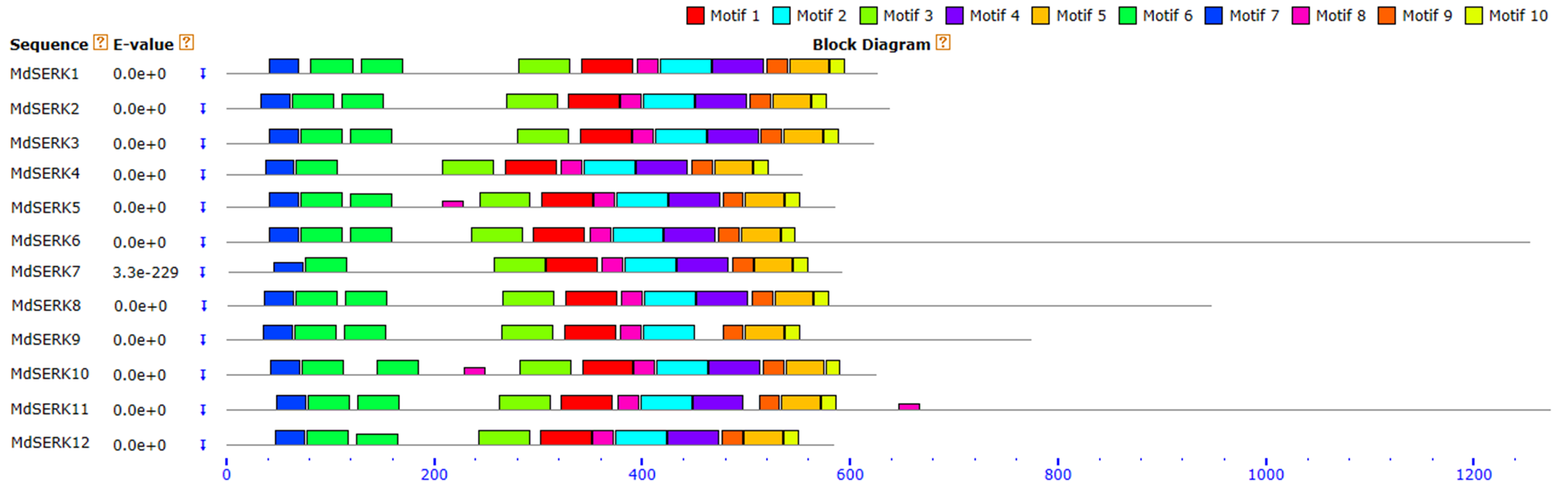

Supplement: Supplementary file 2 — Figure S1. Distribution of conserved motifs among apple SERK family members. Ten putative motifs are indicated with numbers in colored boxes. Names of all members and combined E-values are provided on the left; motif sizes are indicated below. Please see Table S5. for details of motifs. (TIF 540 kb) [file 12864_2018_5342_MOESM2_ESM.tif]

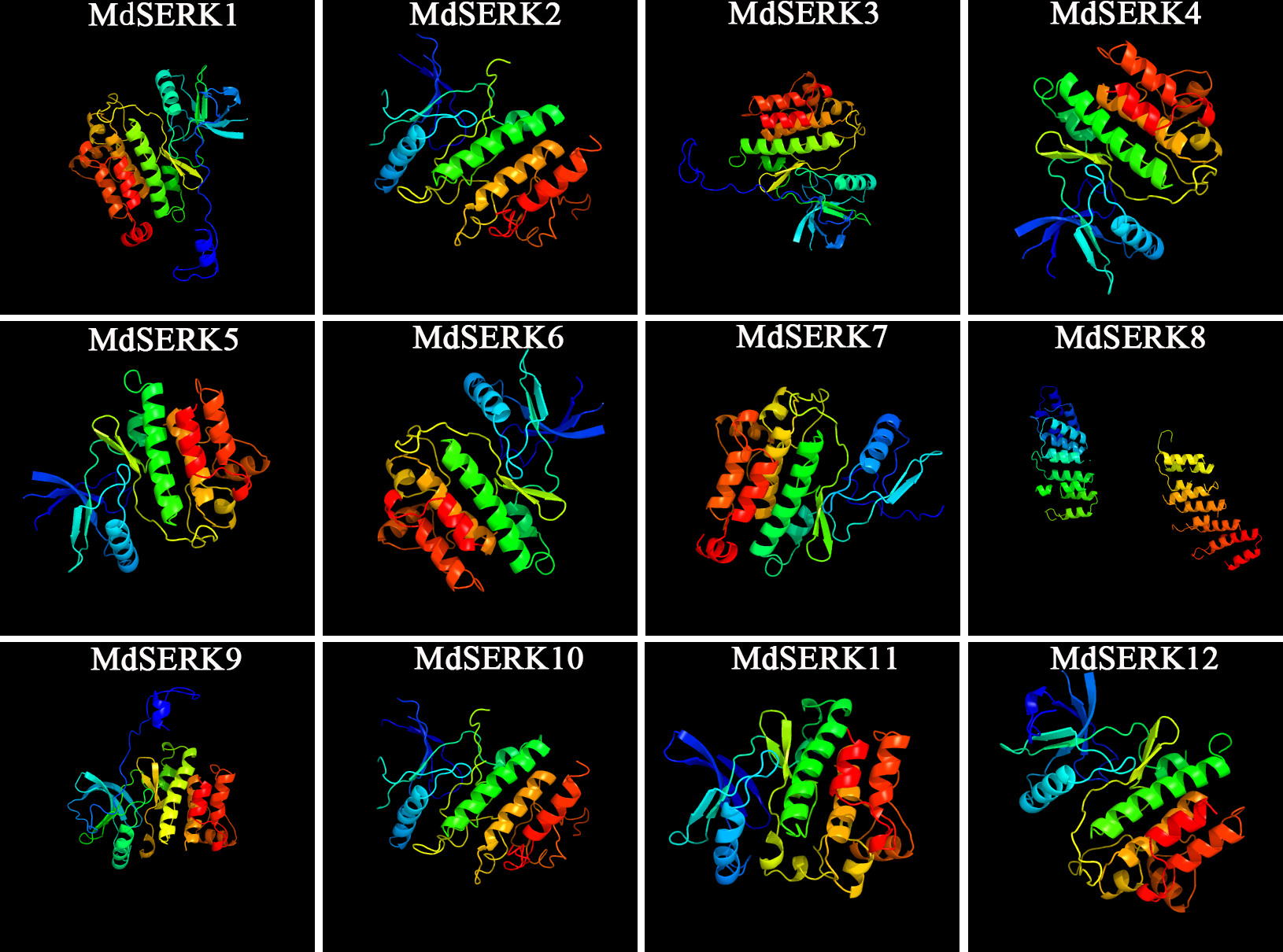

Supplement: Supplementary file 3 — Figure S2. Predicted dimensional structures of MdSERK proteins. (TIF 5808 kb) [file 12864_2018_5342_MOESM3_ESM.tif]

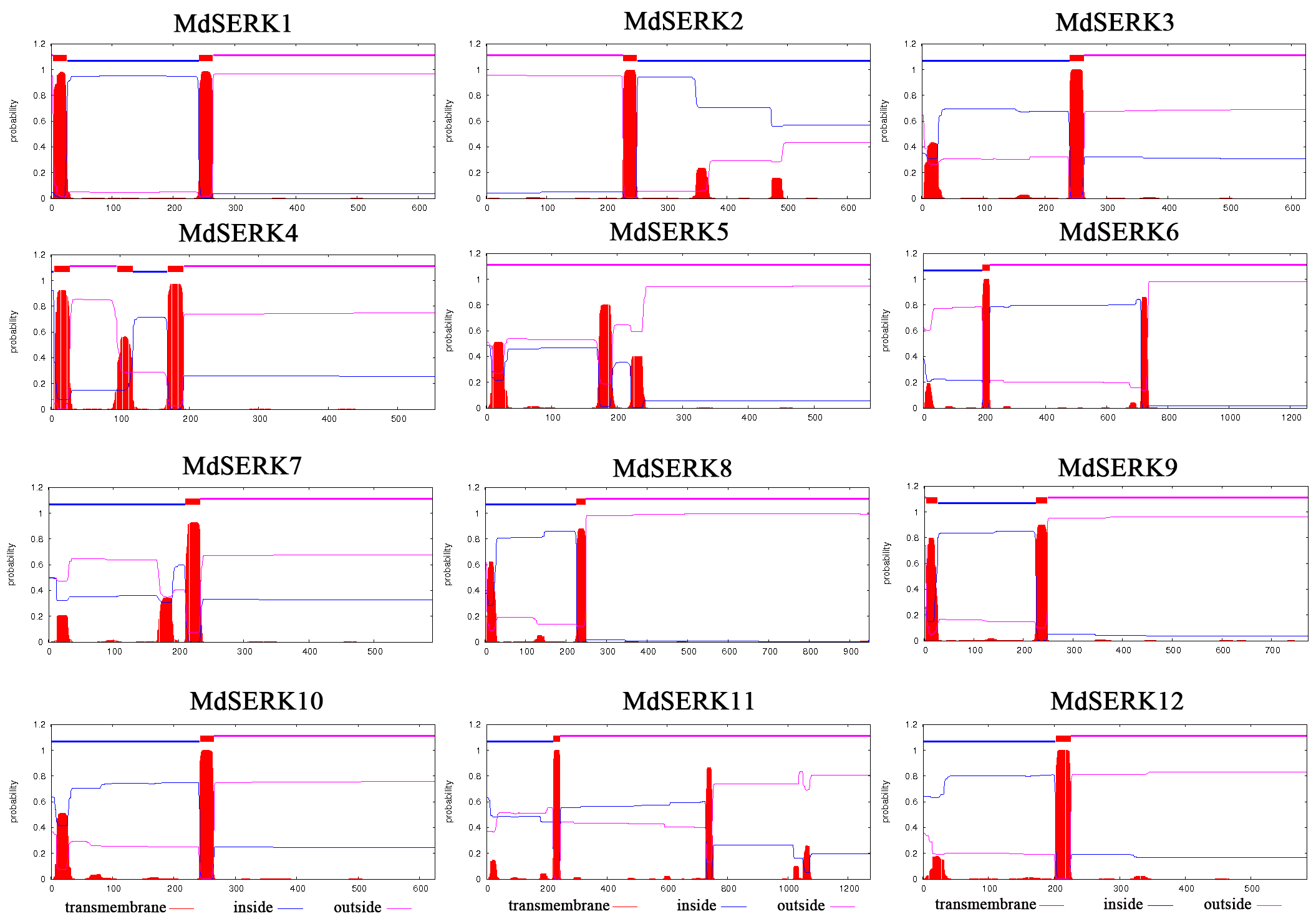

Supplement: Supplementary file 4 — Figure S3. Transmembrane topology analysis of MdSERK proteins. Transmembrane helices of the MdSERK proteins were predicted with TMHMM server v2.0. Red peaks indicate predicted transmembrane helices. (TIF 163 kb) [file 12864_2018_5342_MOESM4_ESM.tif]

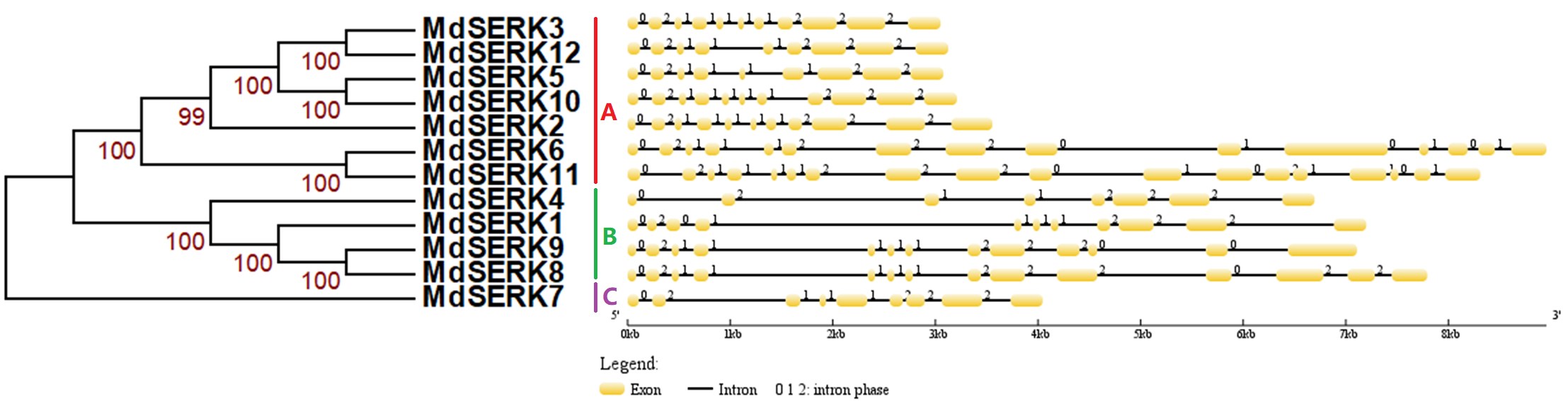

Supplement: Supplementary file 5 — Figure S4. Intron/exon organization of apple SERK genes. Phylogenetic analysis and intron/exon organization of apple SERK genes. Numbers above or below branches indicate bootstrap values. Differently colored areas correspond to genes in each group. (TIF 417 kb) [file 12864_2018_5342_MOESM5_ESM.tif]

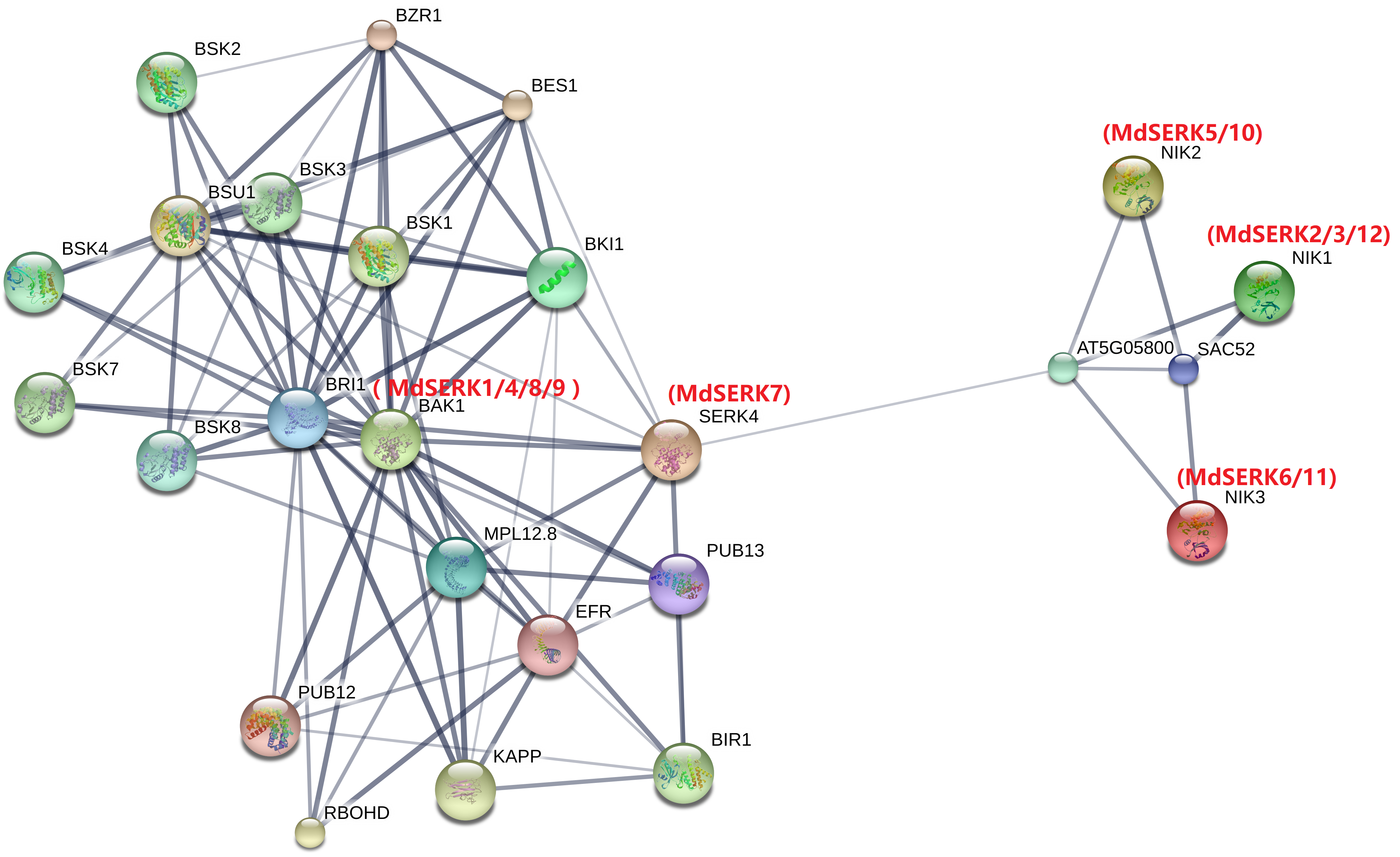

Supplement: Supplementary file 7 — Figure S6. Interaction network for proteins encoded by apple SERK genes according to Arabidopsis thaliana orthologs. Line thickness is related to combined score. Homologous genes in apple are indicated in red font in parentheses. (TIF 1893 kb) [file 12864_2018_5342_MOESM7_ESM.tif]

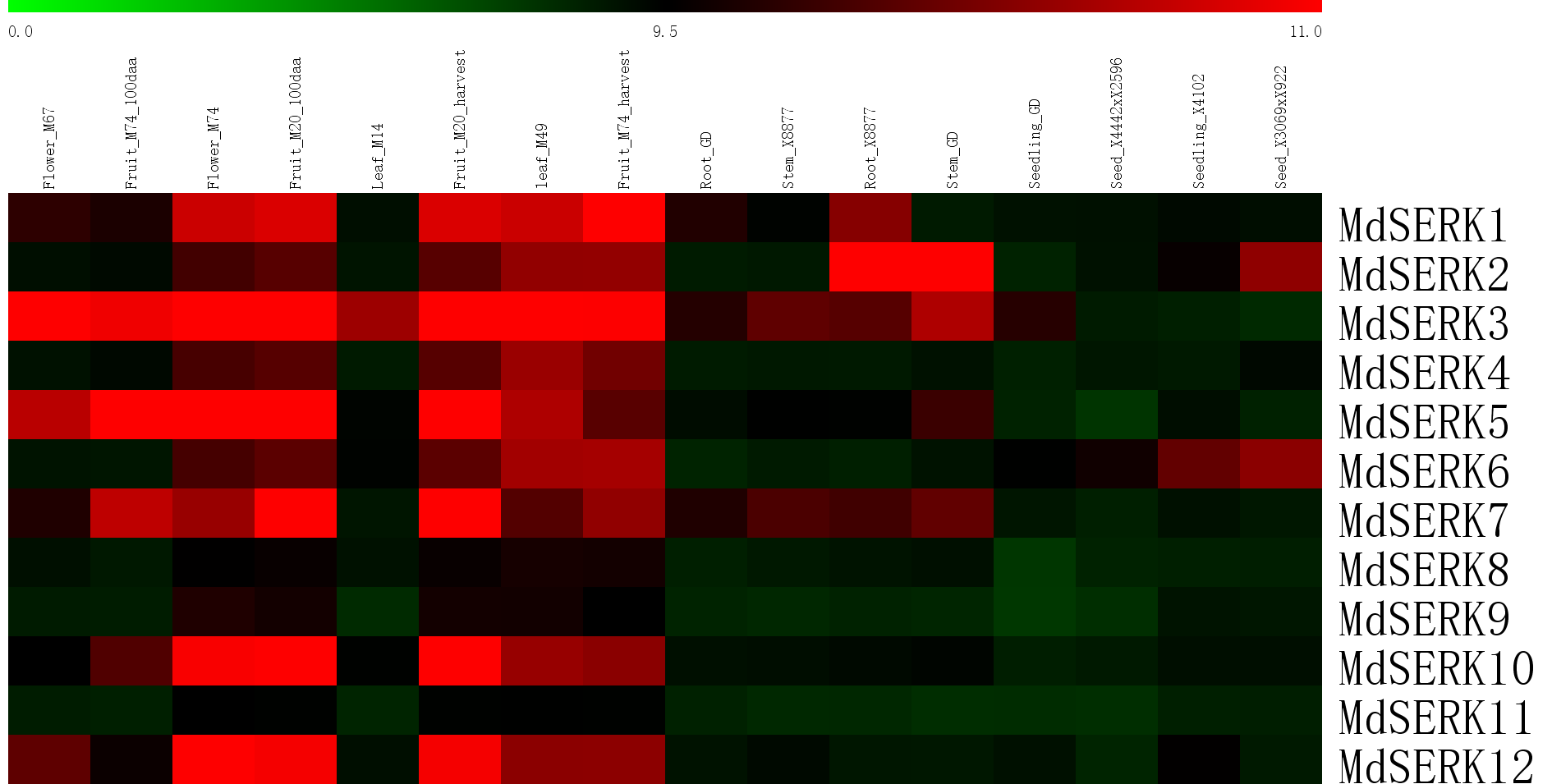

Supplement: Supplementary file 8 — Figure S7. Heat map showing MdSERK gene transcript levels in different tissues. Relative transcript levels are based on ArrayExpress data. (TIF 5289 kb) [file 12864_2018_5342_MOESM8_ESM.tif]
